# Structural Imaging of Native Cryo-Preserved Secondary Cell Walls Reveals the Presence of Macrofibrils and Their Formation Requires Normal Cellulose, Lignin and Xylan Biosynthesis

**DOI:** 10.3389/fpls.2019.01398

**Published:** 2019-10-23

**Authors:** Jan J. Lyczakowski, Matthieu Bourdon, Oliver M. Terrett, Ykä Helariutta, Raymond Wightman, Paul Dupree

**Affiliations:** ^1^Department of Biochemistry, University of Cambridge, Cambridge, United Kingdom; ^2^Natural Material Innovation Centre, University of Cambridge, Cambridge, United Kingdom; ^3^The Sainsbury Laboratory, University of Cambridge, Cambridge, United Kingdom; ^4^Institute of Biotechnology/Department of Biological and Environmental Sciences, University of Helsinki, Helsinki, Finland

**Keywords:** scanning electron microscopy, cell walls, macrofibrils, cellulose, xylan, lignin, softwood, hardwood

## Abstract

The woody secondary cell walls of plants are the largest repository of renewable carbon biopolymers on the planet. These walls are made principally from cellulose and hemicelluloses and are impregnated with lignin. Despite their importance as the main load bearing structure for plant growth, as well as their industrial importance as both a material and energy source, the precise arrangement of these constituents within the cell wall is not yet fully understood. We have adapted low temperature scanning electron microscopy (cryo-SEM) for imaging the nanoscale architecture of angiosperm and gymnosperm cell walls in their native hydrated state. Our work confirms that cell wall macrofibrils, cylindrical structures with a diameter exceeding 10 nm, are a common feature of the native hardwood and softwood samples. We have observed these same structures in *Arabidopsis thaliana* secondary cell walls, enabling macrofibrils to be compared between mutant lines that are perturbed in cellulose, hemicellulose, and lignin formation. Our analysis indicates that the macrofibrils in *Arabidopsis* cell walls are dependent upon the proper biosynthesis, or composed, of cellulose, xylan, and lignin. This study establishes that cryo-SEM is a useful additional approach for investigating the native nanoscale architecture and composition of hardwood and softwood secondary cell walls and demonstrates the applicability of *Arabidopsis* genetic resources to relate fibril structure with wall composition and biosynthesis.

## Introduction

The majority of carbon in terrestrial biomass is stored in forests as wood (Pan et al., 2011; Ramage et al., 2017). The current classification system distinguishes two types of timber. Wood from Angiosperm trees is known as hardwood and the wood made by Gymnosperm species is described as softwood (Ramage et al., 2017). Despite significant differences in tissue organization and chemical composition, both these types of timber are almost entirely formed from plant secondary cell walls—an extracellular matrix made primarily from cellulose, lignin, and hemicelluloses (Schweingruber, 2007). Considering the ecological and industrial importance of wood and other cell wall materials, our knowledge of the exact arrangement of these polymers in the cell wall remains poor. A better understanding of the molecular architecture and ultrastructure of cell walls is needed to describe the complex spatio-temporal deposition pattern of the cell wall polymers. This may contribute to the development of more efficient biofuel feedstocks (Loque et al., 2015), to the improvement in our understanding of novel biomaterials such as nanocellulose (Jarvis, 2018), and to applications such as advanced approaches for the use of timber in the construction industry (Ramage et al., 2017).

Cellulose is the main constituent of plant cell walls (Pauly and Keegstra, 2008). At the molecular level, cellulose has a simple repeating structure of β-1,4-linked glucopyranosyl residues. These glucan chains coalesce to form a crystalline cellulose microfibril. The exact structure of the microfibril is unknown, however, it has been suggested the elementary microfibril consists of 18 or 24 individual glucan chains (Gonneau et al., 2014; Hill et al., 2014; Turner and Kumar, 2017). Individual cellulose microfibrils associate to form larger order structures known as macrofibrils (Niklas, 2004). In plant primary cell walls this close-contact association may be limited to selected parts of the microfibril which is proposed to lead to formation of so-called biomechanical hotspots (Cosgrove, 2014). A range of imaging and spectroscopic techniques has been used to investigate cellulose macrofibrils in secondary cell walls, as reviewed by Purbasha et al. (2009), but due to technical challenges the precise structure in native, unprocessed, hydrated secondary cell walls remains poorly described. Lignin is the main non-polysaccharide component of both hardwood and softwood and is made by coupling of monolignol radicals in secondary cell walls. Three main monolignols exist in plants, which, once turned into chemical radicals by the activity of laccases and peroxidases, can couple in a random manner to form a lignin polymer made from guaiacyl (G), syringyl (S), and p-hydroxyphenyl (H) units (Ralph et al., 2004). The monolignol composition of hardwood and softwood differs, with the former consisting of predominantly S and G units and the latter being made almost solely from G units (Vanholme et al., 2010). The process of lignification is important for wood mechanical properties. *Arabidopsis* mutant plants with reduced lignin content or altered monolignol composition often have collapsed xylem vessels and can be severely dwarfed (Bonawitz and Chapple, 2010). Lignin is proposed to associate with cell wall polysaccharides to form the recalcitrant matrix (Terrett and Dupree, 2019).

Xylan and galactoglucomannan (GGM) are the principal hemicelluloses in hardwood and softwood. Xylan is a polymer of β-1,4-linked xylopyranosyl residues and is the main hemicellulose in hardwood but is also present in softwood (Scheller and Ulvskov, 2010). Hardwood and softwood xylans carry α-1-2 linked glucuronic acid (GlcA) branches which can be methylated on carbon 4 leading to formation of 4-O-Methyl-glucuronic acid (MeGlcA) (Scheller and Ulvskov, 2010). In addition to GlcA and MeGlcA [together, [Me]GlcA] decorations, hardwood xylan hydroxyls are acetylated on carbon 2, carbon 3 or both carbons of the monomer. The softwood xylan, in addition to the MeGlcA branches, carries α-1,3-linked arabinofuranosyl decorations (Scheller and Ulvskov, 2010; Busse-Wicher et al., 2016b). The presence of [Me]GlcA branches on xylan is important for the maintenance of biomass recalcitrance (Lyczakowski et al., 2017) and, together with acetylation in hardwood and arabinose decorations in softwood, these substitutions are mostly distributed with an even pattern on xylosyl units (Bromley et al., 2013; Busse-Wicher et al., 2014; Busse-Wicher et al., 2016b; Martinez-Abad et al., 2017). This so-called “compatible” patterning of xylan substitutions is thought to allow the hydrogen bonding between xylan, in a two-fold screw conformation, and the hydrophilic surface of the cellulose microfibril (Busse-Wicher et al., 2016a; Simmons et al., 2016; Grantham et al., 2017). GGM is the main hemicellulose in softwood (Scheller and Ulvskov, 2010) but is also present in hardwood xylem. GGM has a backbone formed from both β-1,4-linked mannosyl and glucosyl residues with some mannosyl residues substituted by an α-1,6-linked galactosyl branch. The GGM backbone can also be acetylated. The arrangement of mannose and glucose units in softwood GGM is thought to be random, but a recently described regular structure GGM found in *Arabidopsis* mucilage was proposed to bind to both the hydrophilic and hydrophobic surface of the cellulose microfibril (Yu et al., 2018). *In vitro* studies using TEM and 1D ^13^C NMR indicate that a range of branched and unbranched mannan and glucomannan structures can interact with bacterial cellulose (Whitney et al., 1998). Softwood GGM is also proposed to interact with the cellulose microfibril (Terashima et al., 2009) and recent evidence demonstrates that it can form covalent linkages with lignin (Nishimura et al., 2018).

Although we now have a better understanding of secondary cell wall composition and the nature of the interactions between its main constituents, a picture of the ultrastructural assembly of wall polymers into a secondary cell wall matrix is not yet complete. Solid state NMR (ssNMR) analysis has been applied extensively to the study of polymer interactions in both primary and secondary walls. This, for example, provided evidence that in dried primary wall samples from *Arabidopsis*, pectin and xyloglucan may be interacting with the cellulose microfibril (Dick-Perez et al., 2011). Analysis of hydrated secondary cell wall of *Arabidopsis* with solid state NMR indicated that xylan is likely to interact with the hydrophilic surface of the cellulose microfibril as a two-fold screw (Simmons et al., 2016; Grantham et al., 2017). Recent ssNMR analysis indicates that in dried cell walls of grasses, xylan is likely to interact with lignin (Kang et al., 2019). Despite providing excellent insights into the proximity of different cell wall components ssNMR cannot provide information about the assembly of these constituents into higher order structures. Some insights into this process have been achieved with other techniques. This includes application of vibrational microspectroscopy techniques such as FT-IR and Raman to study the orientation of cellulose and other cell wall components in the matrix, as reviewed by Gierlinger (2018). AFM has been applied to the study of cell wall matrix assembly, but the work has been focused on primary cell walls (Cosgrove, 2014) and only recent advances allowed nanoscale resolution imaging of dried spruce secondary cell walls (Casdorff et al., 2017). Moreover, insights into the assembly of cellulose microfibrils in wood walls of conifers (Fernandes et al., 2011) and dicots (Thomas et al., 2014) have been obtained using wide-angle X-ray scattering (WAXS) and small-angle neutron scattering (SANS).

In addition to these various approaches, other studies have attempted to use scanning electron microscopy (SEM) to study the structure of plant cell walls. Low temperature SEM (cryo-SEM), in which the sample is rapidly frozen and then maintained cold during imaging, has been used to study the collapse of pine needle tracheid cell walls upon prior dehydration (Cochard et al., 2004) and to visualize the bulging of root hairs in the *kojak* (cellulose synthase-like) mutant (Favery et al., 2001). Additionally, higher magnification cryo-SEM has been used to visualize cell walls of wheat awns (Elbaum et al., 2008). Some awn cell walls exhibit structural differences that are dependent upon the level of hydration and cryo-SEM revealed extensive layering within the wall, however, the technique was not further optimized to investigate individual fibrils. Field emission (FE) SEM techniques were effectively used to study the alignment of cellulose microfibrils in *Arabidopsis* hypocotyls (Refregier et al., 2004), roots (Himmelspach et al., 2003), and stems (Fujita et al., 2013). FE-SEM has also been applied to investigate wood structure, including observations of microfibril alignment in fixed cell walls of fir tracheids (Abe et al., 1997) and lignin distribution in spruce tracheids (Fromm et al., 2003). Importantly, FE-SEM analysis of dehydrated pine and poplar wood suggests that secondary cell walls of these species contain macrofibrils—cylindrical fibrillar structures with a diameter of up to 60 nm, which presumably comprise of bundles of elementary cellulose microfibrils (Donaldson, 2007). Moreover, the diameter of these macrofibrils was observed to increase with increasing lignification, suggesting that the macrofibrils may be formed from association of lignin and cell wall polysaccharides. This analysis was extended further to wood from Ginkgo where the FE-SEM was combined with density analysis to propose a model of macrofibril formation based on cellulose, GGM, xylan, and lignin interaction (Terashima et al., 2009).

It has been suggested that some of the treatments used in preparation of the FE-SEM cell wall samples have little impact on the microfibril arrangement and that the technique may provide a true representation of native (unprocessed) cell wall features (Marga et al., 2005). The FE-SEM techniques applied to secondary cell wall samples, however, included additional steps such as (i) fixation and exposure to organic solvents (ii) a thermal treatment that may result in some degree of wall degradation (Fromm et al., 2003) and (iii) a thick coating of heavy metal which may impact upon the resolution (Donaldson, 2007), raising questions about the effect these may have on interpretation of the wall structure. Visualization of native, hydrated, secondary cell walls with environmental FE-SEM has been challenging and the resolution of obtained images has been low (Donaldson, 2007). We present here a technique for the analysis of native, fully-hydrated, secondary cell wall material from angiosperm and gymnosperm plant species using cryo-SEM. The use of an ultrathin 3 nm platinum film, together with cryo-preservation at high vacuum, enabled us to demonstrate that cell wall macrofibrils are a common feature in all types of native secondary cell wall material analyzed. Importantly, we were able to detect the presence of macrofibrils in *Arabidopsis thaliana* vessel secondary cell walls. This allowed us to make use of the readily available cell wall-related genetic resources, revealing *Arabidopsis* macrofibril diameter to be dependent upon cellulose, xylan, and lignin.

## Materials and Methods

### Plant Material

*Picea abies*, (spruce) one-year old branch was acquired from 30–50cm tall potted plants grown outdoors purchased from Scotsdale (Great Shelford, Cambridgeshire, UK). *Ginkgo biloba*, (Ginkgo) material, consisting of the narrow ends of branches of diameter approximately 3–5 mm was obtained from 15 year old trees grown at the Cambridge University Botanic Garden. For both spruce and Ginkgo, samples from two individuals were analyzed.

Hybrid aspen (*Populus tremula* x *Populus tremuloides*, clone T89), referred to as poplar in the text, was grown *in vitro* (20°C, with a 16-h light, 8-h dark photoperiod, with illumination at 85 μE m^−2s−1^) during 76 to 80 days after micro-propagation on 1/2MS media with vitamins (Duchefa M0222), 1% sucrose, 0.7% Agar. Samples from three individuals were analyzed. For field grown poplar (*Populus tremula*), material was obtained from one year old branches of two approximately 30 year old individuals grown at the Cambridge University Botanic Garden.

*A. thaliana* (*Arabidopsis*) Columbia-0 ecotype plants were grown in a cabinet maintained at 21°C, with a 16-h light, 8-h dark photoperiod. Stem material was collected from 7-week-old plants. Mutant insertion lines described in published work were used in this study. Specifically, Col-0 ecotype *irx3-7* plants (Kumar and Turner, 2015; Simmons et al., 2016), representing a mutant allele of CESA7, *irx9-1* (Brown et al., 2005), *irx10-1* (Brown et al., 2005), *esk1-5* (Lefebvre et al., 2011; Grantham et al., 2017), *4cl1-1* (Vanholme et al., 2012), *lac4-2* (Berthet et al., 2011) and *csla2-1csla3-2csla9-1* (Goubet et al., 2009) were studied. Mutants of *IRX1* and *IRX5* gene were in Ler ecotype (Taylor et al., 2003). Plants were analyzed alongside the Col-0 or Ler wild type (WT) material. For each genotype three individuals were analyzed.

### Cryo-SEM Sample Preparation and Imaging

**Figure 1:**
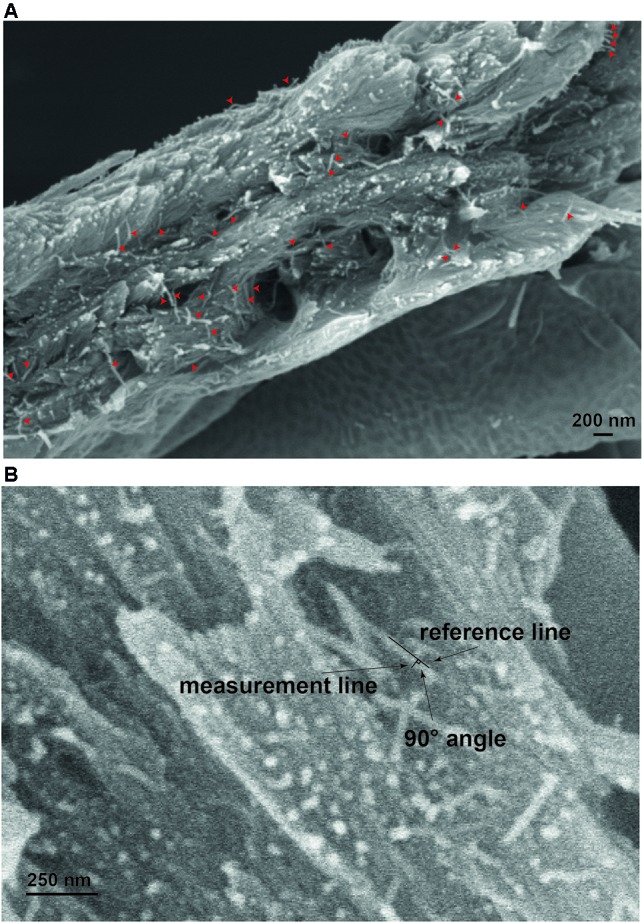
Measurement of cell wall macrofibrils. **(A)** Example of macrofibrils which would be considered for measurement. Only macrofibrils that were resolvable from their neighbors were analyzed. The diameter was measured at a site along the length of the macrofibril and not at the fractured ends. Measurement **(B)** was carried out by placing one line in parallel to the macrofibril and measuring the length of a line perpendicular to it and spanning the width of the structure to be analyzed.

Fresh stems of 7 week old *Arabidopsis* plants were prepared for imaging as outlined in **Figure S1**. Firstly, 1 cm length sections were cut from the bottom part of the stems and mounted vertically in recessed stubs containing a cryo glue preparation consisting of a 3:1 mixture of Tissue-Tec (Scigen Scientific, USA) and Aquadog colloidal graphite (Agar Scientific, Stansted, UK) (see steps 1 to 4 on **Figure S1**). Stem sections were immediately (within 5 min of harvest) plunge frozen in liquid nitrogen slush (step 5 on **Figure S1**), transferred under vacuum, fractured and then coated with 3 nm of platinum (step 6 on **Figure S1**) using a PT3010T cryo-apparatus fitted with a film thickness monitor (Quorum Technologies, Lewes, UK). The short time between freezing and harvesting serves to prevent drying of the sample where only the exposed surface, not the fractured face, is expected to exhibit some water loss during the short time it is exposed to air. Finally, fractured stems were imaged using a Zeiss EVO HD15 Scanning Electron Microscope (step 7 on **Figure S1**) and maintained at −145°C using a Quorum cryo-stage assembly. The electron source is a Lanthanum Hexaboride HD filament. Images were acquired using a secondary electron detector and an accelerating voltage of between 5 and 8 kV with a working distance between 4 and 6 mm. Quantification of the width of cell wall macrofibrils was performed using ImageJ software (Schneider et al., 2012). For the measurements of macrofibril width between 25 and 50 macrofibrils were selected at random on each image analyzed (**Figure 1A**). To quantify the width a line was drawn parallel to the fibril axis. The length of a second line, perpendicular to the fibril axis line and across the width of the macrofibril, was quantified as the macrofibril width (**Figure 1B**). Each fibril width measurement was standardized for the platinum layer applied during the coating process by subtracting the width of the standardized coat from the original measurement. Imaging without the cryo-preservation was performed by visualizing hand sectioned platinum coated specimens with the stage maintained at room temperature. For preparation of these samples all freezing steps were omitted.

### Sampling and Statistical Analysis

For spruce, Ginkgo, and field grown poplar, stem sections were taken from two individual trees and 150 macrofibrils were measured from three tracheids/vessels that had each been coated with platinum separately. Imaging of poplar was performed in technical triplicate from three *in vitro* grown trees and 150 poplar macrofibrils were measured from three separately coated vessels as for the gymnosperm samples. For *Arabidopsis*, cryo-SEM imaging of vessels was carried out on three biological replicates, each from separate individuals. 150 macrofibril diameters were measured across the three individuals.

Statistical analysis was performed using packages available with R software (Team, 2014). Statistical tests, either Student’s T test or ANOVA, used to compare average measurements for samples are defined in Figure legends. The variance between each pairwise combination was estimated to be similar with Levene’s test.

## Results

### Softwood and Hardwood Secondary Cell Walls Contain Macrofibrils

In order to investigate and compare the nanoscale architecture of gymnosperm and angiosperm cell walls we analyzed stem sections taken from spruce, Ginkgo, and poplar using cryo-SEM. Stems were placed in the SEM specimen stub and immediately frozen in nitrogen slush, fractured and then coated with platinum, before being passed in to the SEM chamber for imaging. Nitrogen slush is a suspension of solid nitrogen that enables high freezing rates, greatly reducing the Leidenfrost effect during plunge freezing and thus minimizing structural damage (Sansinena et al., 2012). The fine grain size attributed to platinum sputtering allows small and densely packed objects to be resolved. This rapid sample preparation protocol serves to better maintain sample hydration levels and native structures for optimal EM imaging in a high vacuum environment.

**Figure 2:**
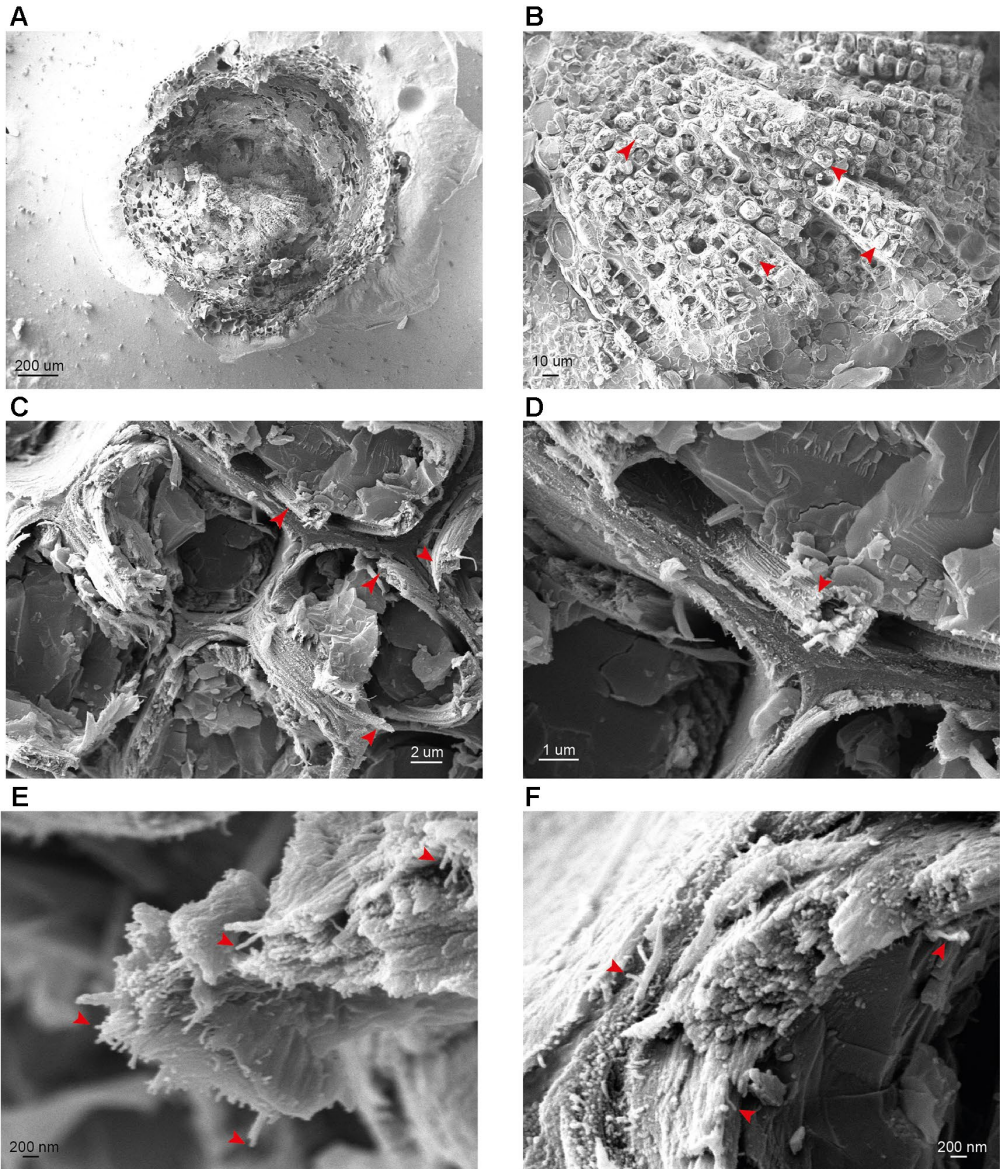
cryo-SEM analysis of spruce stem sections. **(A**–**F)** Representative images of stem sections of one-year-old spruce branch at different magnifications. Red arrows indicate tracheids **(B)**, macrofibril bundles **(C** and **D)** and individual macrofibrils **(E** and **F)**. Scale bars are provided for each image.

We first investigated whether our cryo-SEM protocol gave comparable results to the previous FE-SEM analysis of both softwood and hardwood secondary cell walls (Donaldson, 2007). To examine if macrofibrils are found in natively hydrated, non-pretreated cell walls, cryo-SEM imaging was performed on unprocessed, frozen softwood and hardwood samples. For observing gymnosperm cell wall architecture, we first prepared softwood samples from spruce and used a low magnification to see an overview of stem cross-section (**Figure 2A**) and tracheid structure (**Figure 2B**). The inner part of the stem cross section was composed of densely packed xylem tracheids, each surrounded by cell walls. To investigate the appearance of the secondary cell walls, higher magnification images of these parts of tracheid cells were acquired. This enabled us to observe that the tracheid cell walls contain fibrous structures which frequently assembled into larger aggregates (**Figures 2C, D**, red arrows). After a further increase in magnification, individual fibrils became resolvable (**Figures 2E, F**) and their diameter was found to exceed the 3 nm diameter calculated for a single softwood elementary microfibril (Fernandes et al., 2011). Therefore the observed fibrils, if composed of cellulose, represent a higher order structure that fits the description of a “macrofibril” (Niklas, 2004; Donaldson, 2007). Similarly to spruce stem, sections from another gymnosperm, the Ginkgo, were also observed to contain macrofibrils (**Figure S2**). These data show that, in line with previously reported SEM imaging of dried, processed plant material (Donaldson, 2007; Terashima et al., 2009), the native, hydrated cell walls of spruce and Ginkgo also contain macrofibrils. Therefore, these structures may contribute to the higher order assembly of native gymnosperm cell walls.

**Figure 3:**
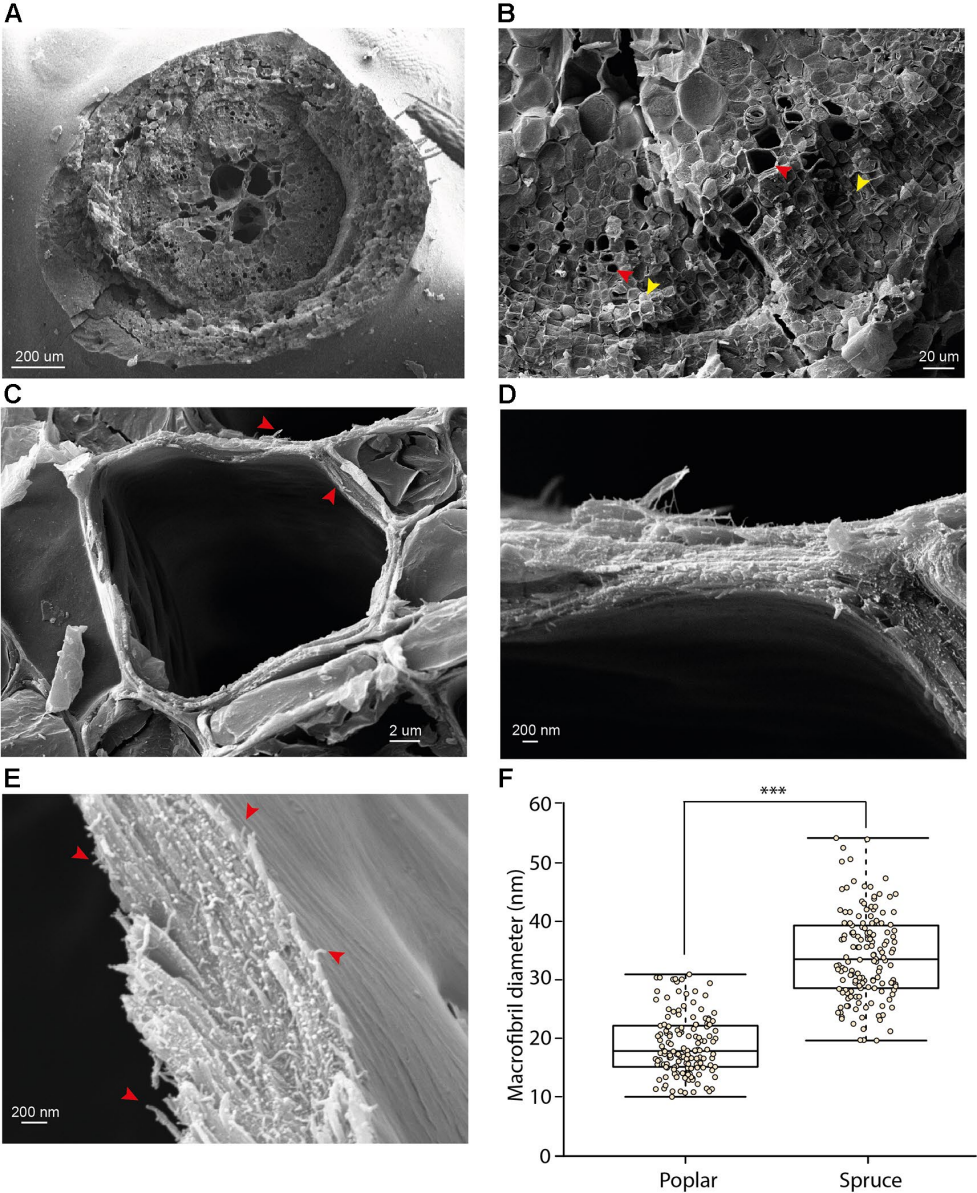
cryo-SEM analysis of poplar stem sections **(A**–**E)** Representative images of stem sections of *in vitro* grown poplar trees at different magnifications. Red arrows show vessels **(B)** and macrofibrils **(C** and **E)**. Yellow arrows indicate fiber cells **(B)**. Higher magnification images **(C**, **D** and **E)** are presented for vessels. Scale bars are provided for each image. **(F)** Diameter of spruce tracheid cell wall fibrils compared to these observed in poplar vessel cell walls. For each bar 150 individual fibrils were measured. Boxplots mark the median and show between 25^th^ and 75^th^ percentile of the data. *** denotes p ≤ 0.00001 in Student’s t-test.

We extended the analysis to the model hardwood species, poplar. Vessels, a distinct cell type of hardwood xylem, were clearly visible using low magnification (**Figures 3A, B**). In addition to the vessels, xylem fiber cells were also observed (**Figure 3B**; red and yellow arrows for vessels and fiber cells respectively). For some cells we were able to observe spiral thickenings which were preserved during sample preparation and extended above the surface of the fracture plane (**Figure 3B**). We focused upon the vessel cell walls which showed clearly visible fibrous structures at a vessel-to-vessel boundary (**Figure 3C**). Analysis of vessel cell walls at a higher magnification revealed a clear presence of macrofibril structures, similar to those observed in spruce, in the poplar samples (**Figures 3D, E**). To investigate the dimensions of the macrofibrils we measured their diameter in poplar and spruce (**Figure 3F**). Our measurements are broadly similar to those reported in a previous study (Donaldson, 2007). We carried out comparative analysis of macrofibril diameter between hardwood and softwood by measuring 150 individual macrofibrils in poplar, spruce and Ginkgo. While the diameter of spruce and Ginkgo macrofibrils was not significantly different (**Figure S3**), the diameter of macrofibrils in poplar secondary cell walls was significantly smaller than that of spruce macrofibrils (**Figure 3**). Spruce and Ginkgo were grown in the field while poplar samples were obtained from *in vitro* grown plants. To control for this difference in growth conditions we also analyzed samples from field grown poplar trees. There was no statistically significant difference in the macrofibril diameter between the two poplar samples (**Figure S4**). For both hardwood and softwood we observed variation in the macrofibril diameter. This may reflect biological differences or may be a result of technical challenges associated with macrofibril width measurement.

### *Arabidopsis* Secondary Cell Wall Macrofibrils Contain a Cellulose Scaffold

**Figure 4:**
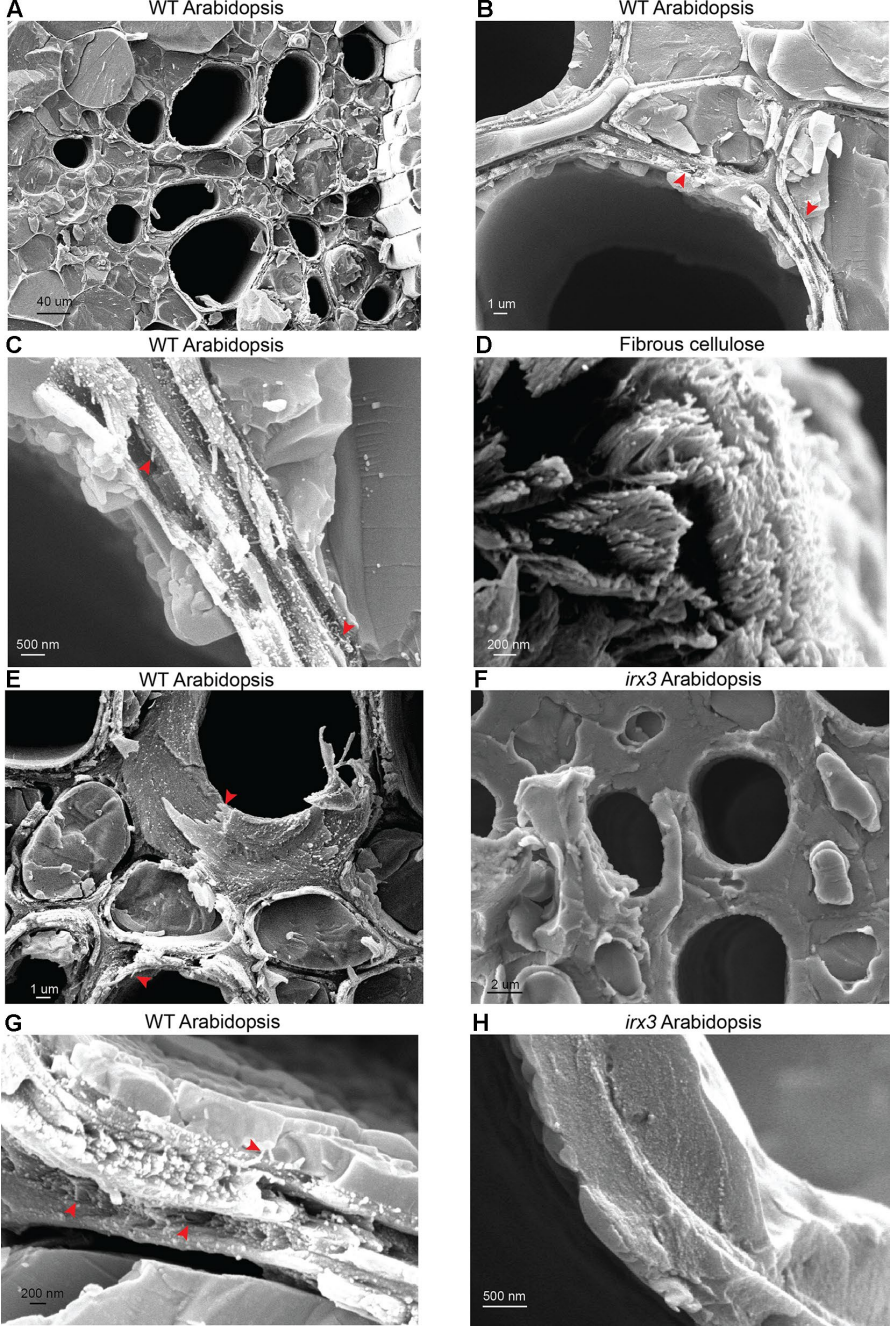
Analysis of *Arabidopsis* stem sections and fibrous cellulose. **(A-C)** Imaging of WT vessels at increasing magnification **(D)** Imaging of fibrous cellulose standard from cotton linters shows cell wall fibrils with an appearance similar to structures seen *in planta*. **(E)** Imaging of individual vessels in WT plants. **(F)** Imaging of individual vessels in *irx3* plants. **(G**, **F)** Macrofibrils are detectable in WT *Arabidopsis* and are absent in *irx3* secondary cell walls. Red arrows indicate the macrofibril structures throughout the figure. Scale bars are provided for each image.

To further evaluate the nanoscale architecture of plant cell walls and identify possible constituents of the cell wall macrofibrils, the high magnification cryo-SEM imaging was used to analyze wild type (WT) *Arabidopsis* secondary cell walls (**Figure 4**). The initial analysis investigated the structure of WT xylem vessels (**Figures 4A, B**). Sets of vessel bundles were detected and, using higher magnification, fibrous structures similar to those observed in spruce and poplar were also visible in the fractured *Arabidopsis* material. The width of WT *Arabidopsis* macrofibrils was comparable to that of poplar macrofibrils but not spruce and suggests *Arabidopsis* macrofibrils could be used as a good structural model for hardwoods (**Figures S3** and **S4**). Despite the use of ultra-thin platinum coating, the use of SEM without the cryo-preservation steps did not allow us to observe the *Arabidopsis* macrofibrils with good resolution (**Figure S5**) highlighting the critical importance of sample cryo-preservation to resolve a native cell wall ultrastructure.

Based on the data available in the literature, we hypothesized that the macrofibrils may be mostly composed of cellulose (Fahlen and Salmen, 2002; Donaldson, 2007). To investigate this, and to understand the nature of these macrofibrils further, we performed a comparative analysis between WT vessel cell walls (**Figure 4C**) and a commercially available fibrous cellulose standard (**Figure 4D**) extracted from cotton linters and consisting of 99% pure cellulose (Sczostak, 2009). In this experiment, clear individual fibrils with distinct bright termini were observed in both samples indicating that the vessel wall macrofibrils have a similar appearance to the cellulose fibrils present in this polysaccharide standard. To determine whether these macrofibrils are dependent upon the proper production of cellulose, the morphology of WT *Arabidopsis* vessel cell walls (**Figures 4E, G**) was compared to that of the *irx3* mutant (**Figures 4F, H**). IRX3 is one of three CESA proteins that make up the secondary wall cellulose synthase complex and *irx3* plants are almost completely devoid of cellulose in their secondary cell walls, but not primary cell walls (Taylor et al., 1999). As previously reported, *irx3* plants had collapsed vessels (**Figure S6**), since secondary cell wall cellulose contributes to vessel wall strength (Turner and Somerville, 1997). Interestingly, the *irx3* stems lacked the fibrous structures in their vessel secondary cell walls and, in contrast to WT, the *irx3* cell walls were formed from a largely amorphous matrix (**Figure 4F**). It is likely that this matrix is composed of xylan and lignin, which can still be deposited in the secondary cell wall in the absence of IRX3 activity (Takenaka et al., 2018). Some structures which may resemble cellulose fibrils were present in the primary cell walls of *irx3* plants (**Figure S6**). To further support these observations we analyzed the cell walls of plants mutated in *IRX1* and *IRX5*, encoding other members of the secondary cell wall cellulose complex (**Figure S7**). Similar to *irx3*, the *irx1*, and *irx5* plants lacked fibril-type structures in their cell walls. Taken together, the data show that macrofibril formation is dependent upon cellulose production.

### Reduction in Cell Wall Xylan and Lignin, but Not in GGM Content Decreases the Dimensions of *Arabidopsis* Macrofibrils

**Figure 5:**
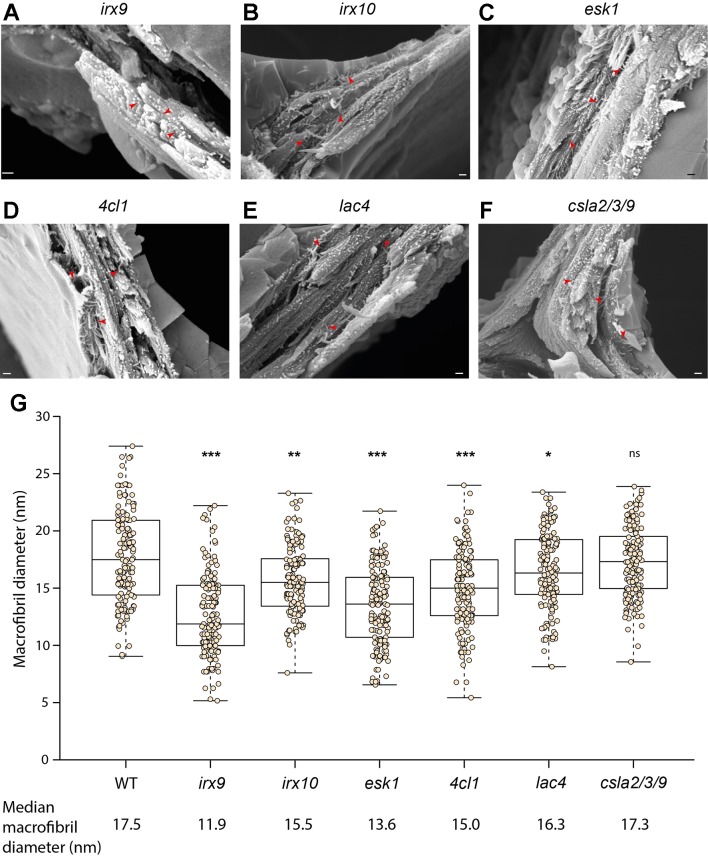
Analysis of macrofibrils in mutant *Arabidopsis* plants. Representative image of **(A)** *irx9*, **(B)** *irx10*, **(C)** *esk1*, **(D)** *4cl1*, **(E)** *lac4* and **(F)** *csla2/3/9 Arabidopsis* macrofibrils. Scale bar corresponds to 200 nm on each image. Red arrows show macrofibrils **(G)** Quantification of macrofibril diameter in WT and mutant *Arabidopsis* plants. N = 150. Boxplots mark a median and show between 25^th^ and 75^th^ percentile of the data. *** denotes p ≤ 0.00001, ** denotes p ≤ 0.0001, * denotes p ≤ 0.05 in Tukey test following ANOVA when compared to WT, ns indicates lack of statistically significant difference. Additional images of each genotype are shown in **Figures S8–S13**.

To investigate the role of xylan in macrofibril formation, cryo-SEM was used to visualize the secondary walls from *irx9*, *irx10*, and *esk1 Arabidopsis* plants (**Figures 5A** and **S8**, **5B** and **S9**, **5C** and **S10**). IRX9 and IRX10 are required for proper xylan synthesis and mutations in the corresponding genes lead to cell wall weakening and collapse of xylem vessels in the *Arabidopsis* model (Brown et al., 2005; Bauer et al., 2006; Brown et al., 2007). The *irx9* plants have impaired xylan synthesis resulting in a decrease of xylan by more than 50% compared to WT (Brown et al., 2007). In *irx10* plants the reduction in xylan content is smaller and does not exceed 20% (Brown et al., 2009). Macrofibrils are clearly observed in *irx9* and *irx10 Arabidopsis* (**Figures 5A, B**). However, the median macrofibril diameter between WT and *irx9* cell wall fibers showed a ∼30% reduction in the xylan synthesis mutant (**Figure 5G**). The median macrofibril diameter of *irx10* plants was ∼10% smaller than that of WT *Arabidopsis* (**Figure 5G**). Although there was a wide variation in macrofibril diameter within each genotype, the difference between the WT macrofibril diameter and the one quantified for the two mutants is statistically significant, suggesting that xylan is incorporated along with cellulose to generate the normal macrofibril size. To investigate the role of xylan–cellulose interaction in the macrofibril formation we assessed the macrofibril size in the *esk1 Arabidopsis* mutant (**Figure 5C**). Mutation in the *ESK1* gene results in reduction of xylan acetylation, but not in a decrease in xylan quantity (Xiong et al., 2013), which leads to changes in xylan [Me]GlcA patterning and loss of interaction between xylan and the hydrophilic surface of the cellulose microfibril (Grantham et al., 2017). In line with the results observed for *irx9* and *irx10* plants the loss of xylan–cellulose interaction caused a reduction in the macrofibril diameter (**Figure 5G**).

Previous work in softwood suggested that lignin (Donaldson, 2007) and GGM (Terashima et al., 2009) may be involved in macrofibril formation. To investigate the role of these two cell wall components in the maintenance of macrofibril structure we performed imaging of *4cl1* (**Figures 5D** and **S11**), *lac4* (**Figures 5E** and **S12**) and *csla2/3/9* (**Figures 5F** and **S13**) mutant *Arabidopsis* cell walls. Both 4CL1 and LAC4 are involved in lignin biosynthesis and plants mutated in genes encoding these enzymes have a 30% and 15% reduction in lignin content respectively (Berthet et al., 2011; Li et al., 2015). The median macrofibril diameter for both *4cl1* and *lac4* was significantly smaller than that calculated for WT (**Figure 5G**). Importantly, the extent of the reduction in macrofibril diameter was in line with the decrease in the lignin content observed for the two mutants, with *4cl1* macrofibrils being ∼15% smaller than the WT ones and *lac4* macrofibrils having ∼7% reduction in the median diameter. Proteins from the CSLA family are involved in the biosynthesis of a hemicellulose GGM and mutations in *csla2/3/9* leads to nearly complete loss of stem GGM in the *Arabidopsis* model (Goubet et al., 2009). Our quantitative analysis indicates that the diameter of macrofibrils of *csla2/3/9 Arabidopsis* was not significantly different to that of the WT plants (**Figure 5G**).

## Discussion

The native nanoscale architecture of woody plant secondary cell walls remains poorly understood due to the challenges of keeping the sample hydrated, which is incompatible with some types of techniques. Studies that analyze dehydrated and fixed plant cell wall samples with FE-SEM (Donaldson, 2007), together with other work which includes SANS experiments investigating spruce (Fernandes et al., 2011) and bamboo samples (Thomas et al., 2015), suggest there is a higher order arrangement of cellulose microfibrils in plant secondary cell walls. Our work reports the application of a cryo-SEM based analysis technique which, using exclusively samples that have not been dried, heated or chemically processed, indicates that secondary cell wall cellulose microfibrils are likely to come together to form larger macrofibril structures. Our study strongly suggests that these structures, at least in the model plant species *A. thaliana*, are sensitive to changes in xylan and lignin.

Previous studies investigated the presence and diameter of macrofibrils in dehydrated softwood samples (Donaldson, 2007). In line with results presented in our work, Donaldson did observe macrofibrils in cell walls of pine tracheids. Moreover, also in agreement with the results presented here (**Figure S4**), these softwood macrofibrils were larger than those seen in hardwoods. In softwood, in addition to various patterned types of xylan (Busse-Wicher et al., 2016b; Martinez-Abad et al., 2017), most of which are likely to be compatible with binding to the hydrophilic surface of the cellulose fibril, the cell walls contain large quantities of acetylated GGM (Scheller and Ulvskov, 2010) which may contribute to macrofibril width. Indeed, gymnosperm GGM was proposed to interact with the cellulose microfibril in cell walls of Ginkgo (Terashima et al., 2009). Therefore, the significant difference in macrofibril diameter observed between hardwood and softwood samples may be due to the differences in the cell wall composition. Consequently, we hypothesize that in gymnosperms, GGM, along with xylan, may contribute to the macrofibril size in a way similar to what we observed for xylan in *Arabidopsis* macrofibrils. With an average diameter ranging between 20 and 34 nm, the size of pine macrofibrils measured by Donaldson was somewhat smaller than that measured in spruce wood in the current work. However, these observations are not necessarily inconsistent. Donaldson dehydrated the wood samples prior to the SEM imaging. As the spacing between bundled softwood cellulose microfibrils, estimated to be equal to 3 nm by small angle neutron scattering, is sensitive to wood hydration levels (Fernandes et al., 2011), at least part of the difference in the macrofibril diameter might be due to the changes in the water content within the sample analyzed with SEM. Interestingly, Donaldson reported that macrofibrils in dried poplar wood, depending on their position in cell wall, have an average diameter ranging from 14 to 18 nm, which is similar to what was measured for both poplar and *Arabidopsis* as a part of our study. This observation suggests that the softwood macrofibril size may be more sensitive to drying than the hardwood one. This in turn suggests that, in addition to compositional disparities, hydration could contribute to the differences in softwood and hardwood macrofibril characteristics. In addition to providing scientific insight, this result highlights that imaging of the cryo-preserved secondary cell walls offers significant advance over the previously used techniques.

Interestingly, similar to a previous report (Donaldson, 2007), we observed that macrofibrils in both hardwood and softwood have a range of diameters. The reasons for this variation in size are not clear. It is possible that the number of individual cellulose microfibrils that come together to form the macrofibril structure in both hardwood and softwood is not constant. This may be regulated by coordinated movement of CesA complexes or their density during cell wall synthesis (Li et al., 2016). It was proposed that the macrofibril diameter is proportional to the degree of cell wall lignification (Donaldson, 2007), which may also vary between the structures. This hypothesis is supported by our results which indicate that the cell wall lignin content influences macrofibril diameter in *Arabidopsis*. Variations may also originate from environmental conditions. For example, it was shown that wood density may vary correlatively with climate change (Bouriaud et al., 2005). Although much of this effect is likely to be due to cell size and wall thickness, it can be hypothesized that change in wood density may also originate from compositional changes that impact macrofibril assembly and ultrastructure. It would therefore be relevant to assess macrofibrils of perennial trees with samples spanning several years of growth. We cannot rule out that the width variance may originate from the technical limitations of resolving the macrofibrils by SEM. It will be interesting to see if the emerging He-ion technologies, with an increase in resolution and less dependence upon metal coating, reduce this variance (Joens et al., 2013). The cryo-SEM techniques developed as part of our study offer a significant advantage over the previous investigation (Donaldson, 2007) which applied a thicker coat of chromium (mostly 12 nm) that yield films with coarser grains than the thinner (3 nm) platinum films used in our work. Thus, taking the results described by Donaldson and our technological improvements into consideration, we believe that the variance in the macrofibril width observed in both studies is likely to reflect natural material variation.

The prominence of macrofibril structures in *Arabidopsis* cell walls is a surprising discovery of this study. Previously published results using AFM analysis indicate the presence of some bundled microfibrils in primary cell walls of *Arabidopsis* but the extent of this bundling is lower than what was observed in primary cell wall samples from other species (Zhang et al., 2016). AFM is not yet technically feasible for analysis of bundling of hydrated secondary cell walls although recent technical advances allowed visualization of dried spruce wood at a nanometer resolution (Casdorff et al., 2017). The observation of the macrofibrils by cryo-SEM in *Arabidopsis* allowed us to determine the contribution of cellulose, xylan, lignin, and GGM to macrofibril formation, thanks to the availability of secondary cell wall related mutants in this model. Macrofibrils were completely absent in vessel cell walls of *irx1*, *irx3*, and *irx5* plants, which lack secondary cell wall cellulose, indicating that proper cellulose biosynthesis is required for formation and assembly of secondary cell walls polymers into macrofibrils. In addition, we observed that vessel macrofibril diameter is significantly decreased in *irx9, irx10*, and *esk1* plants, suggesting that xylan may also participate in the correct assembly of such structures. While in *irx9* and *irx10* reduction in macrofibril diameter may be associated with decrease in the xylan content the ∼25% reduction in the median macrofibril diameter observed for *esk1 Arabidopsis* is harder to explain. Hardwood xylan is proposed to interact with the hydrophilic surface of the cellulose microfibril as a two-fold screw (Simmons et al., 2016; Busse-Wicher et al., 2016a), and this interaction is facilitated by the even pattern of the [Me]GlcA and acetyl branches on the xylan backbone which is lost in *esk1* plants (Grantham et al., 2017). Therefore, the decrease in macrofibril diameter observed in *esk1 Arabidopsis* indicates that xylan–cellulose interaction may have a role in spacing or proper coalescence of microfibrils to form the elementary macrofibril. It is unclear why the macrofibril diameter is reduced in *esk1*, but perhaps fewer elementary fibrils are incorporated into each macrofibril when xylan is not interacting with the hydrophilic surface of the cellulose fibril. This may be different to the effect observed in flax where the absence of xylan may lead to aggregation of glucan chains into larger fibers (Thomas et al., 2013). Such difference may be associated with variations in the stoichiometry of the cellulose synthase complex which were recently reported for angiosperms (Zhang et al., 2018).

In addition to implicating xylan in the process of macrofibril formation our results indicate that lignin may contribute to assembly of the structures. As such, our results use genetic assignment to extend previous work which has correlated macrofibril diameter with the degree of wall lignification (Donaldson, 2007). Interestingly, we observed that the macrofibril diameter does not correlate with the cell wall GGM content. This may be associated with low abundance of GGM in angiosperms where the polysaccharide accounts for only up to 5% of the cell wall material (Scheller and Ulvskov, 2010). Alternatively, this result may indicate that in *Arabidopsis* GGM might be not involved in macrofibril formation. GGM may play a more significant role in the macrofibril assembly in gymnosperms where it accounts for up to 30% of the cell wall material. Importantly, all our conclusions are based on the analysis of native, hydrated, cell wall samples. The assignment of cell wall macrofibril composition, in their native state, would be impossible using techniques such as immunogold due to the pre-treatment steps needed before the antibody labelling.

In conclusion, our analysis indicates that *Arabidopsis* vessel cell walls contain fibrous structures composed of cellulose and likely contain xylan and lignin. These structures are present in both hardwood and softwood and have a diameter larger than a single cellulose microfibril. Therefore, these structures can be described as cell wall macrofibrils. The reduction in macrofibril diameter observed in *esk1 Arabidopsis* suggests that the interaction between xylan and the hydrophilic surface of the cellulose microfibril may be involved in the assembly of these structures. Therefore, this xylan–cellulose interaction may be important for the maintenance of plant cell wall ultrastructure and mechanical properties (Simmons et al., 2016). The techniques developed here and the discovery of the ubiquitous presence of macrofibrils in hardwood and softwood in their native state will contribute to a better understanding of cell wall assembly processes. Furthermore, the ability to resolve macrofibrils in *Arabidopsis*, along with the availability of genetic resources in this model, will offer the community a valuable tool to further study the complex deposition of secondary cell walls polymers and their role in defining the cell wall ultrastructure. The assembly of cell wall macrofibrils is likely to influence the properties of wood, such as density, which may vary due to different stimuli such as tree fertilization (Makinen et al., 2002) or environmental changes (Bouriaud et al., 2005). Therefore, we expect that the methodology described here will enable to correlate the native nanoscale features of the cell walls, such as the macrofibril diameter, or a specific macrofibril patterning within the cell wall, with wood properties. Consequently, our approach may be useful to assess this aspect of wood quality at a new level and could benefit numerous industries ranging from building construction, paper manufacturing and biofuel production to generation of novel biomaterials such as nanocrystalline cellulose.

## Data Availability Statement

All quantitative datasets generated and analyzed for this study are presented on graphs included in the article/**Supplementary Material**.

## Author Contributions

JL designed the study, conducted the experiments, analyzed the data, and wrote the paper. MB performed poplar imaging experiments, analyzed the data, and wrote the paper. OT analyzed the data and wrote the paper. YH contributed to data analysis and manuscript preparation, RW designed the study, conducted experiments, analyzed the data, and wrote the paper. PD designed the study and contributed to data analysis and manuscript preparation.

## Funding

This work was supported by the Leverhulme Trust Centre for Natural Material Innovation. Analysis of *Arabidopsis* mutant plants was supported as part of The Center for Lignocellulose Structure and Formation, an Energy Frontier Research Center funded by the U.S. Department of Energy (DOE), Office of Science, Basic Energy Sciences (BES), under Award # DE-SC0001090. JL was in receipt of a studentship from the Biotechnology and Biological Sciences Research Council (BBSRC) of the UK as part of the Cambridge BBSRC-DTP Programme (Reference BB/J014540/1). JL is currently supported by a grant from the National Science Centre, Poland as part of the SONATINA 3 programme (project number 2019/32/C/NZ3/00392). MB is employed in YH’s team through the European Research Council Advanced Investigator Grant SYMDEV (No. 323052). YH laboratory is funded by the Finnish Centre of Excellence in Molecular Biology of Primary Producers (Academy of Finland CoE program 2014-2019) (decision #271832); the Gatsby Foundation (GAT3395/PR3); the National Science Foundation Biotechnology and Biological Sciences Research Council grant (BB/N013158/1); University of Helsinki (award 799992091), and the European Research Council Advanced Investigator Grant SYMDEV (No. 323052). OT was a recipient of an iCASE studentship from the BBSRC (Reference BB/M015432/1). The cryo-SEM facility at the Sainsbury Laboratory is supported by the Gatsby Charitable Foundation. The open access publication fees are paid for by the RCUK.

## Conflict of Interest

The authors declare that the research was conducted in the absence of any commercial or financial relationships that could be construed as a potential conflict of interest.

## Abbreviations

1D, one dimensional; AFM, atomic force microscopy; CesA, Cellulose synthase; cryo-SEM, low temperature scanning electron microscopy; FE-SEM, field emission scanning electron microscopy; FT-IR, Fourier-transform infrared spectroscopy; GGM, galactoglucomannan; He-ion, Helium ion; IRX, irregular xylem; [Me]GlcA, methylated and unmethylated form of glucuronic acid; NMR, nuclear magnetic resonance; SANS, small angle neutron scattering; TEM, transmission electron microscopy; WAXS, wide angle x-ray scattering.
