# Supplementary material for: Structural Imaging of Native Cryo-Preserved Secondary Cell Walls Reveals the Presence of Macrofibrils and Their Formation Requires Normal Cellulose, Lignin and Xylan Biosynthesis
Source: Front Plant Sci. 2019 Oct 23;10:1398. doi: 10.3389/fpls.2019.01398 (PMC6819431; doi:10.3389/fpls.2019.01398)
Supplement: Supplementary file 1 [file DataSheet_1.pdf]

Supplementary Material

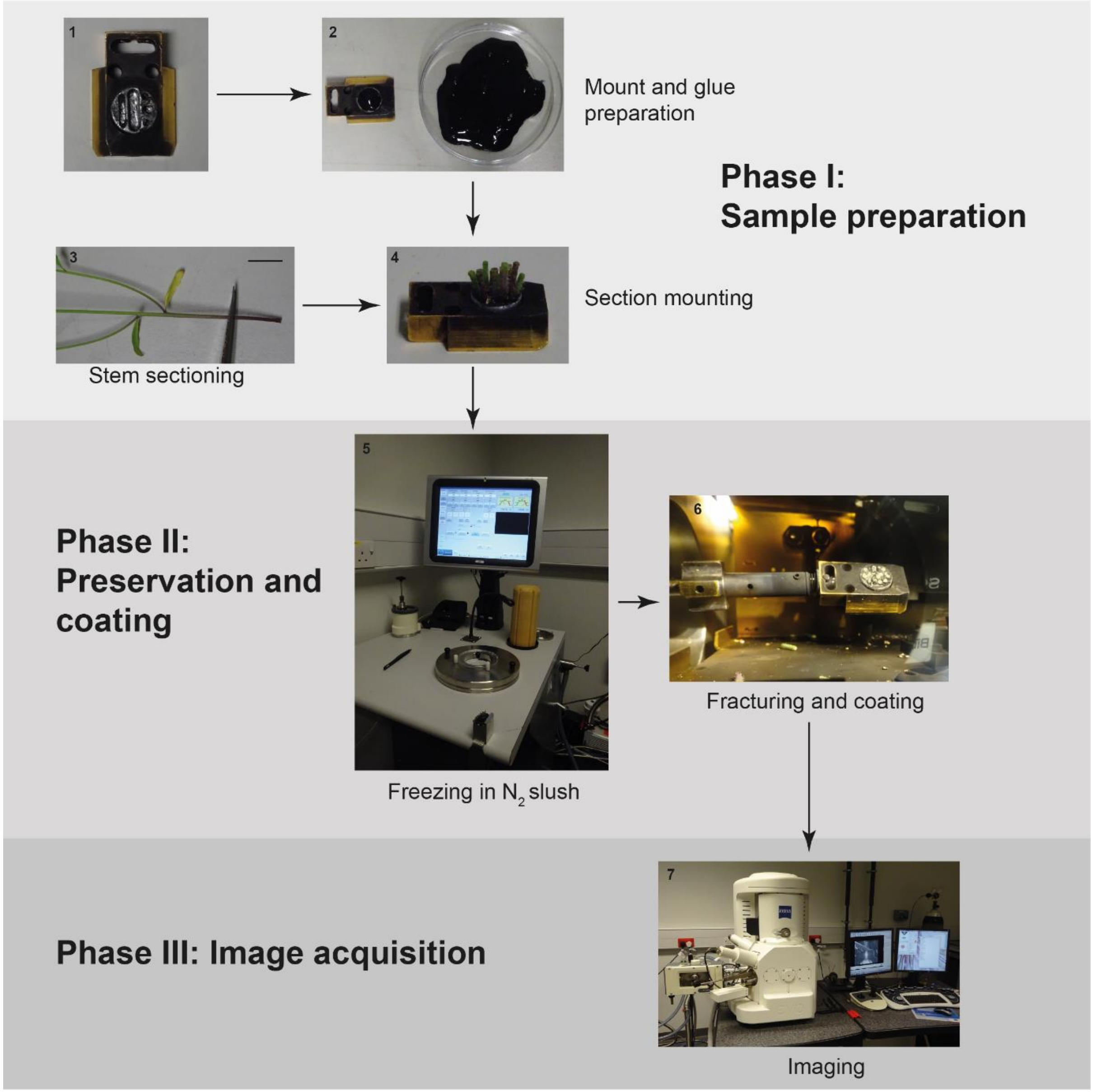

Figure S1. **Overview of the cryo-SEM procedure.** Presented images demonstrate sample preparation required for Arabidopsis imaging. Same protocol was applied for other samples analysed. Step 3 size bar is 1 cm long.

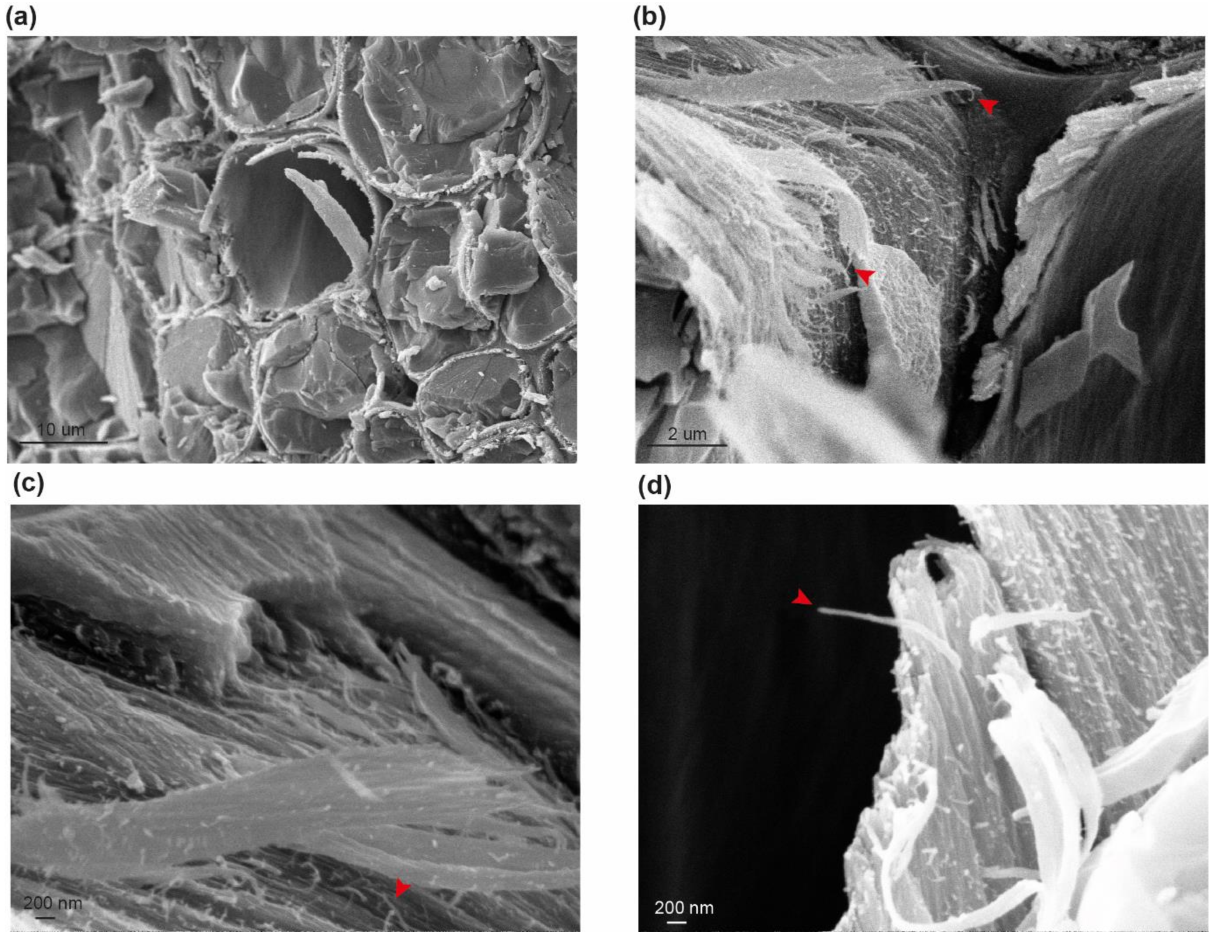

Figure S2. **Cryo-SEM analysis of Ginkgo cell walls**. (a) to (d) shows representative images at different magnification from analysis of stem sections from Ginkgo branches. Red arrows indicate macrofibrils. Size bar is provided for each image.

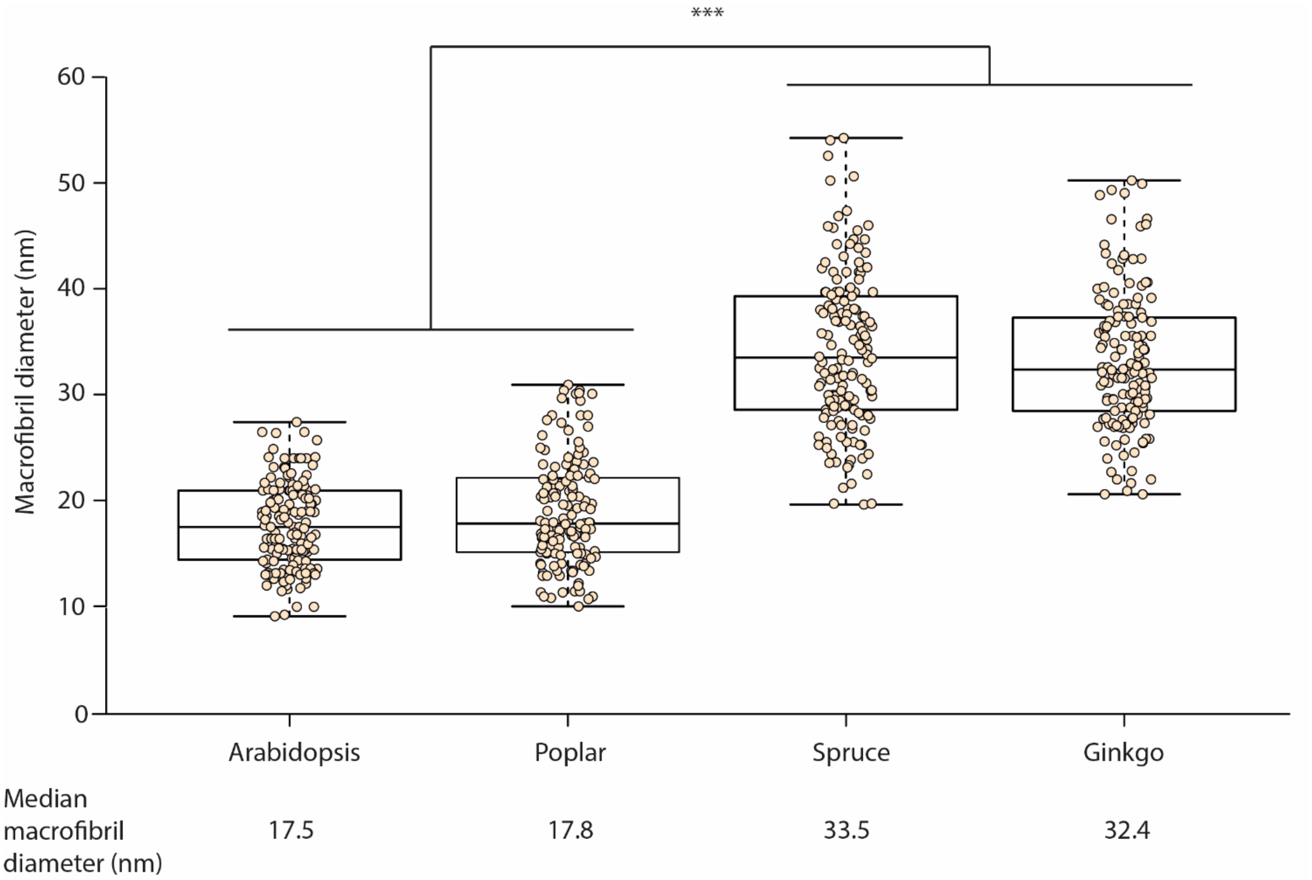

Figure S3. **Comparison of macrofibril diameter in Arabidopsis, poplar, spruce and Ginkgo.** N = 150 macrofibrils. Boxplots mark a median and show between 25th and 75th percentile of the data. *** denotes $p \leq 0.00001$ in Tukey test following ANOVA. No statistically significant difference was observed for the Arabidopsis- poplar and spruce-Ginkgo pairs.

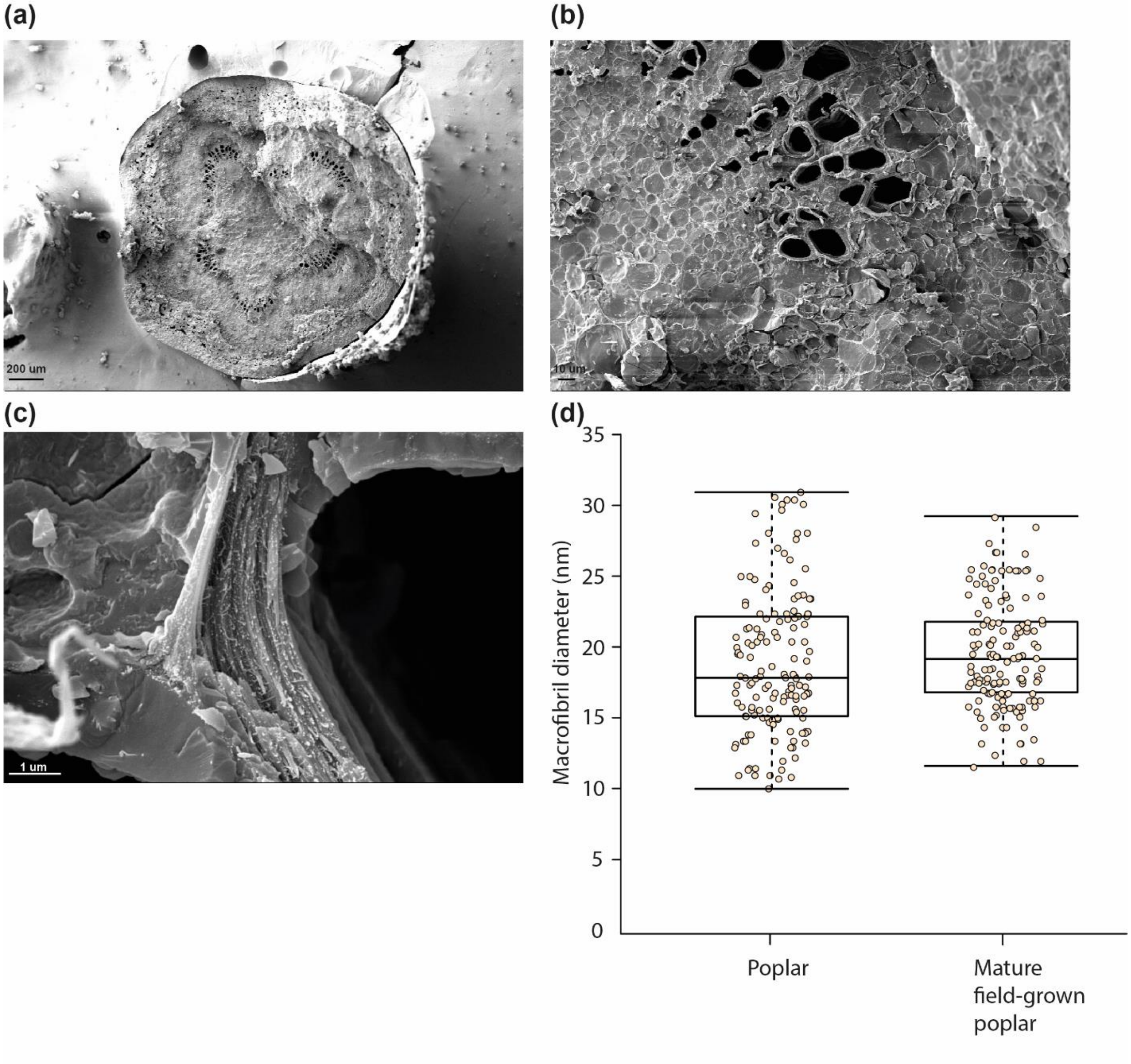

Figure S4. **Imaging of macrofibrils in field grown poplar**. (a)-(c) representative images of poplar (*Populus tremula*) hardwood from sample isolated from a mature tree grown at the Cambridge University Botanic Garden. Size bars are provided for each image. (d) Comparison of macrofibril diameter in *in vitro* and field grown poplar. N = 150 macrofibrils. Boxplots mark a median and show between 25th and 75th percentile of the data. No statistically significant difference was observed for the pair.

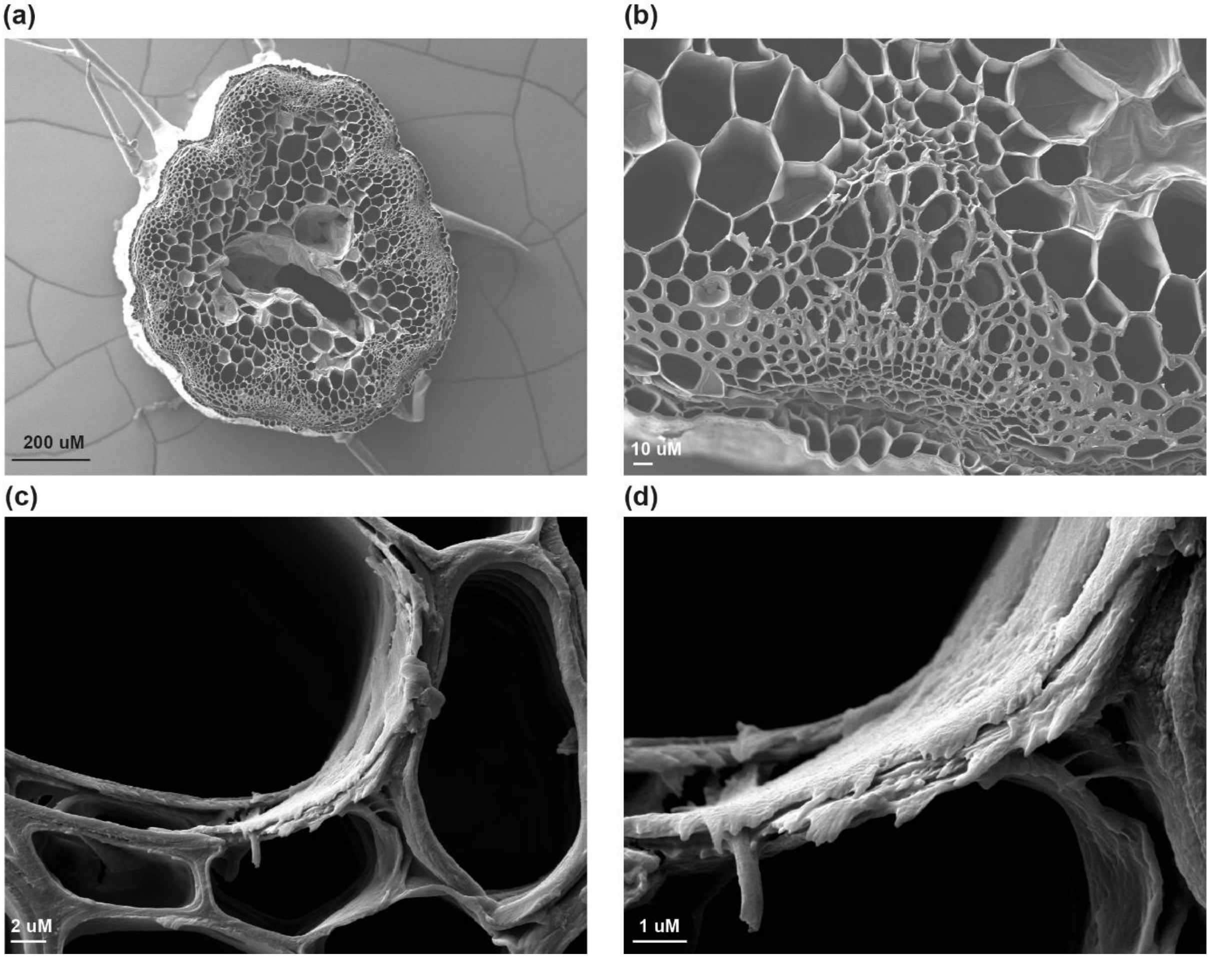

Figure S5. **Analysis of native Arabidopsis samples without the cryo-preservation protocol.** (a)-(d) Representative images of WT Arabidpsis stems imaged without the cryo-preservation protocol. Size bar is provided for each image.

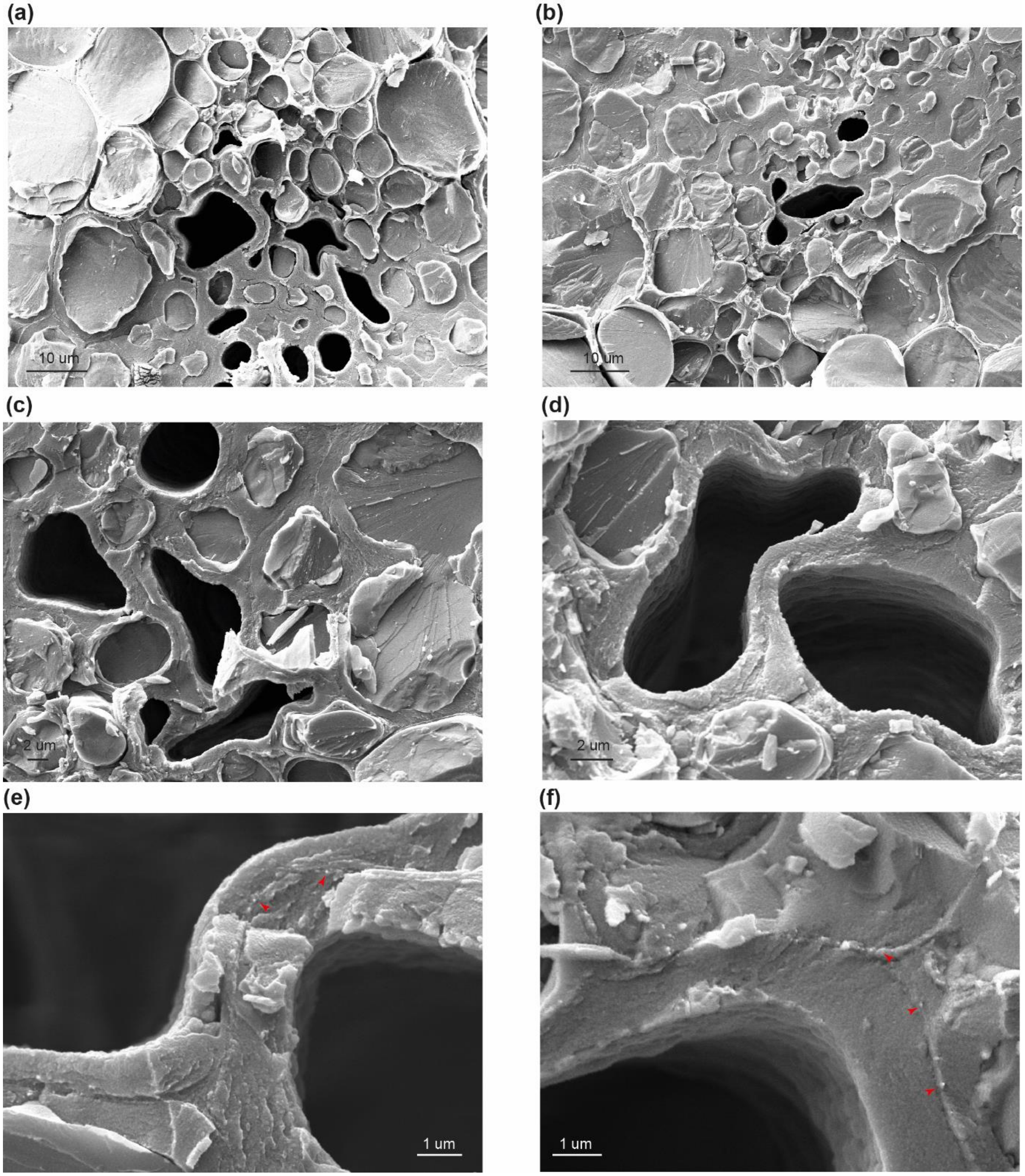

Figure S6. **Cryo-SEM analysis of vessel collapse and primary cell wall cellulose in *irx3* Arabidopsis plants**. (a) to (d) shows vessel collapse on representative images at different magnification from analysis of stem sections from irx3 plants. Size bar is provided for each image. (e) and (f) show putative primary cell wall cellulose structures marked with red arrows.

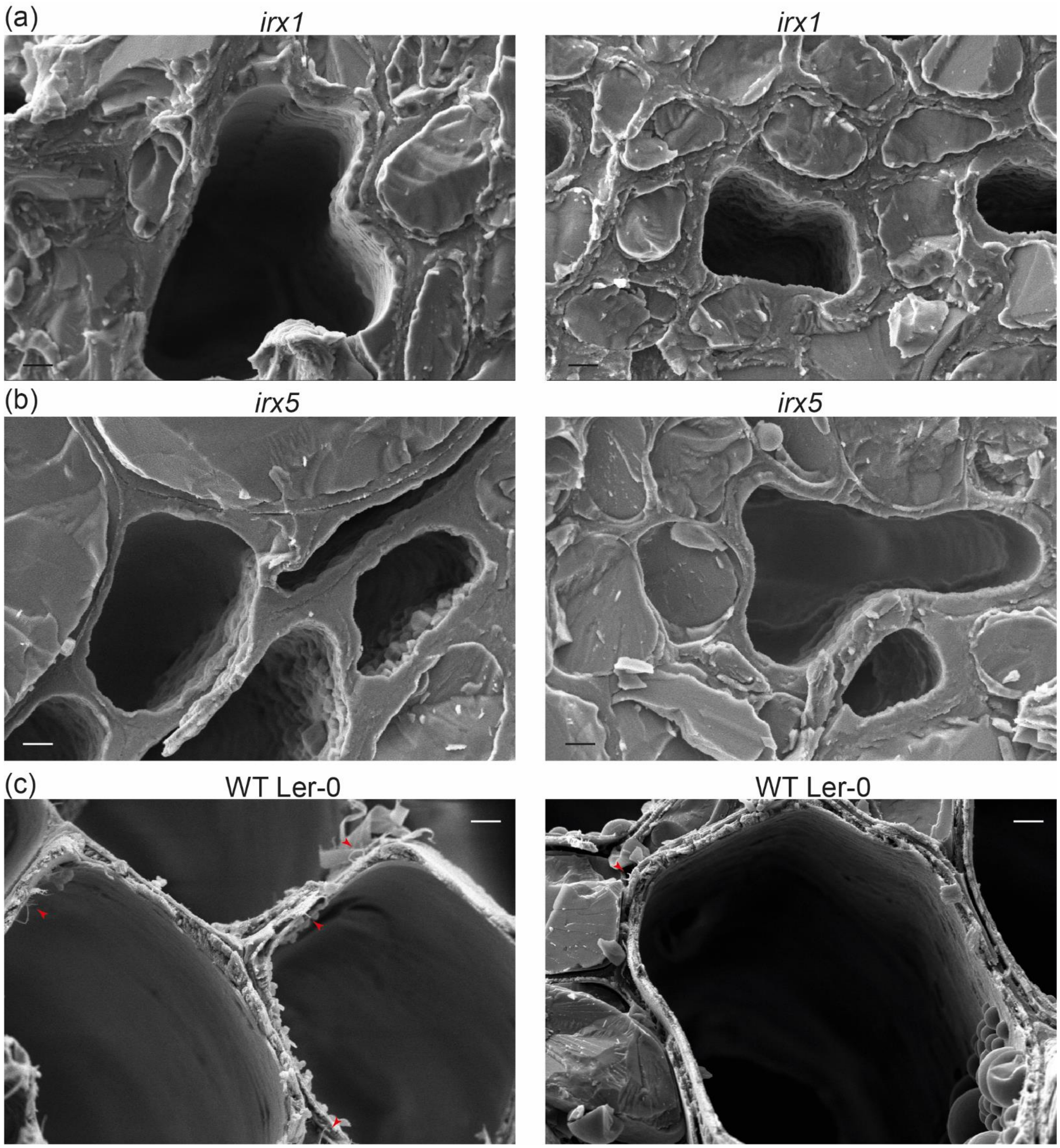

Figure S7. **Cryo-SEM analysis of cell wall structure in Ler *irx1*, *irx5* and WT Arabidopsis plants**. Images showing cell walls of *irx1* (a), *irx5* (b) and WT (c) Ler plants. Size bars provided correspond to 2 µm on all images.

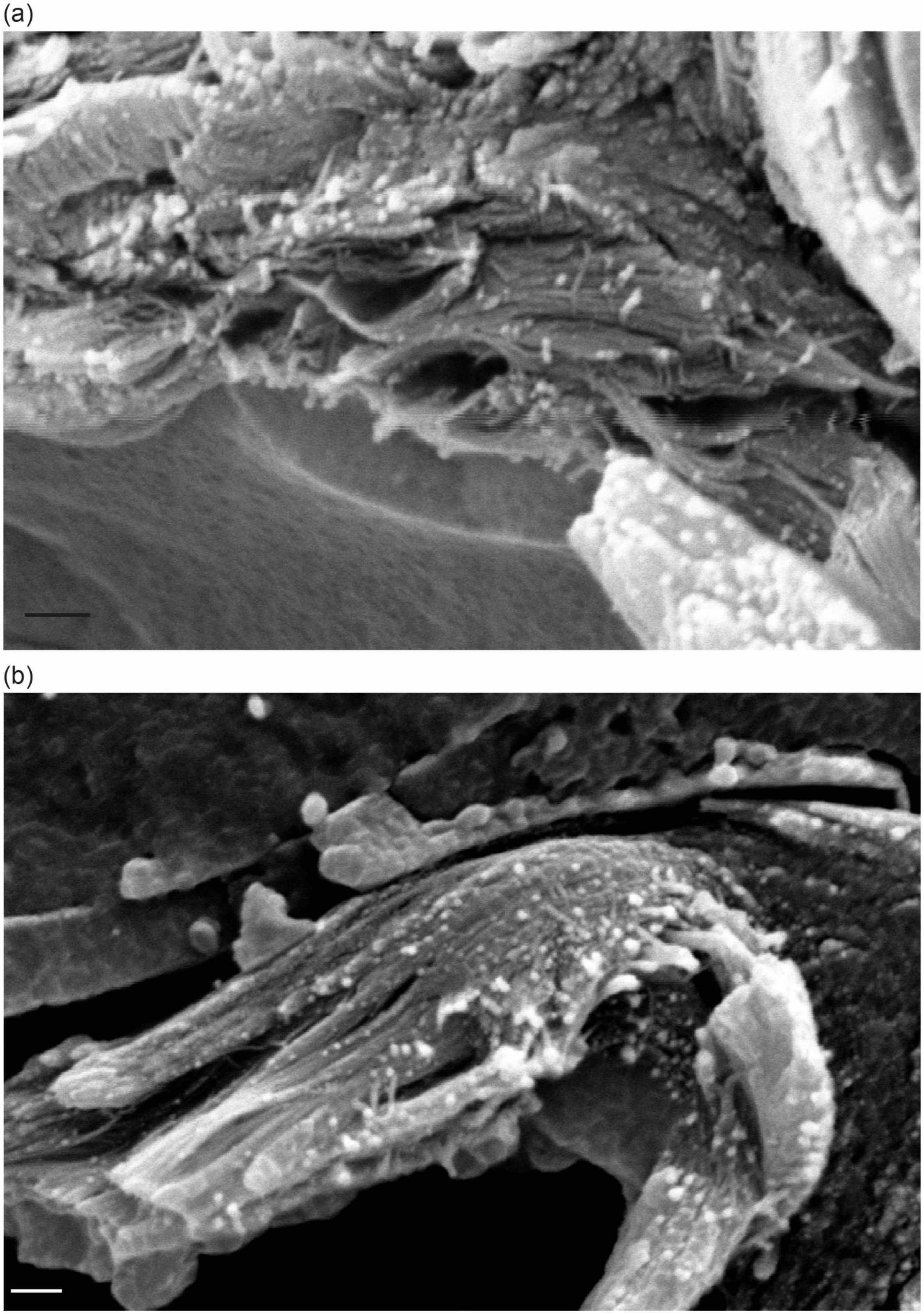

Figure S8. **Further images of cell wall macrofibrils in *irx9* plants**. Images showing cell walls of *irx9* (a and b) are provided. Size bars provided correspond to 200 nm on both images.

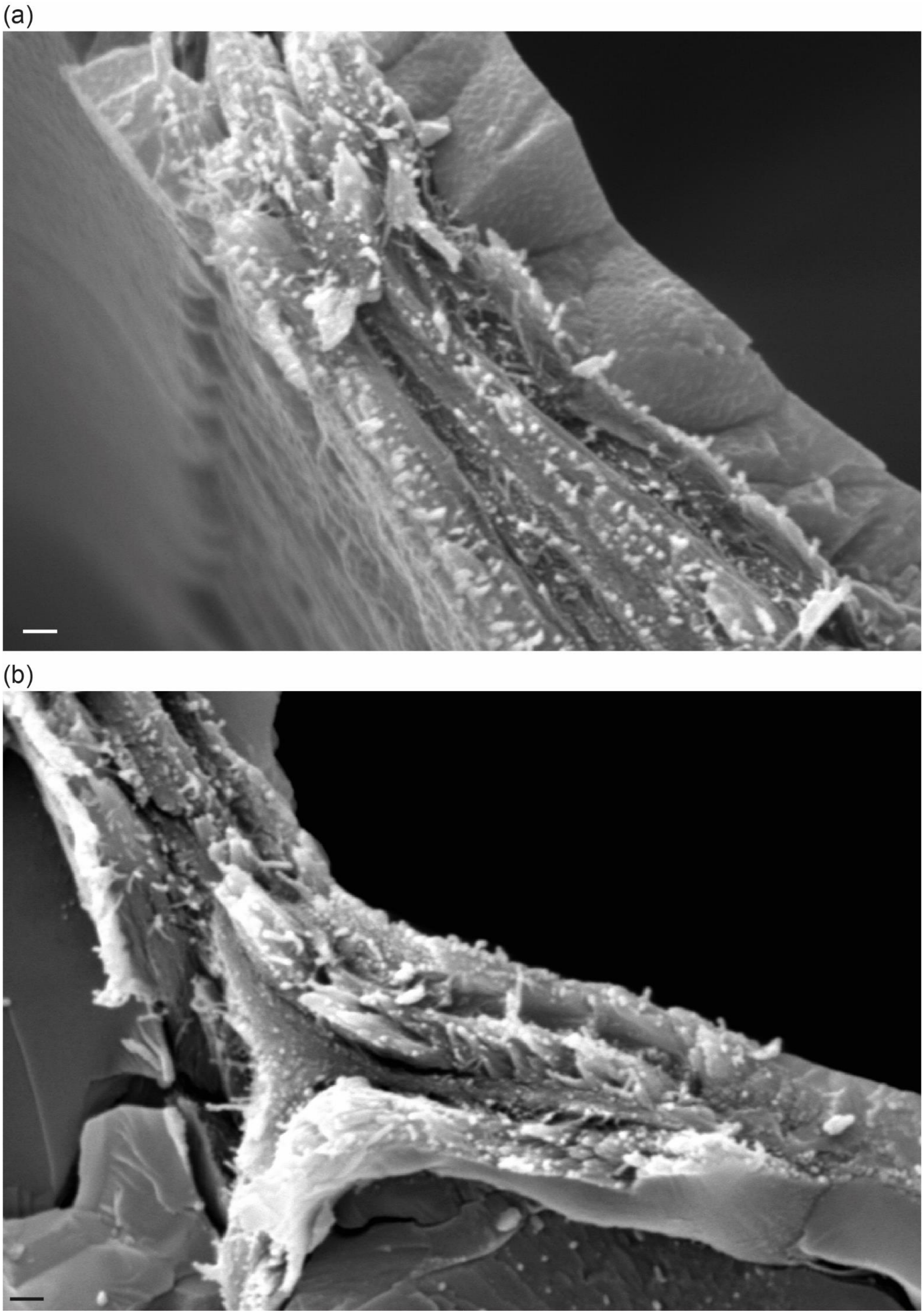

Figure S9. **Further images of cell wall macrofibrils in *irx10* plants**. Images showing cell walls of *irx10* (a and b) are provided. Size bars provided correspond to 200 nm on both images.

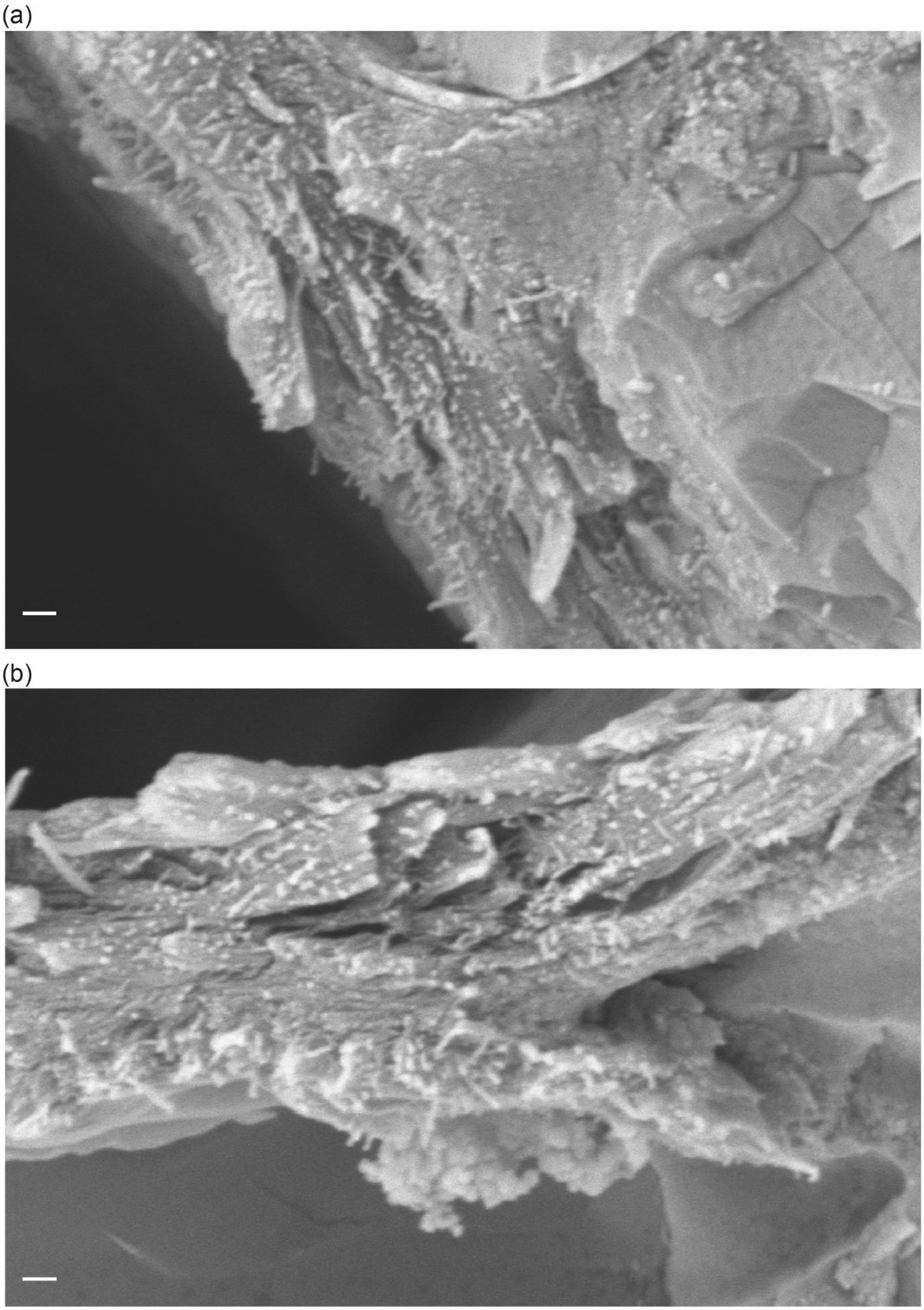

Figure S10. **Further images of cell wall macrofibrils in *esk1* plants**. Images showing cell walls of *esk1* (a and b) are provided. Size bars provided correspond to 200 nm on both images.

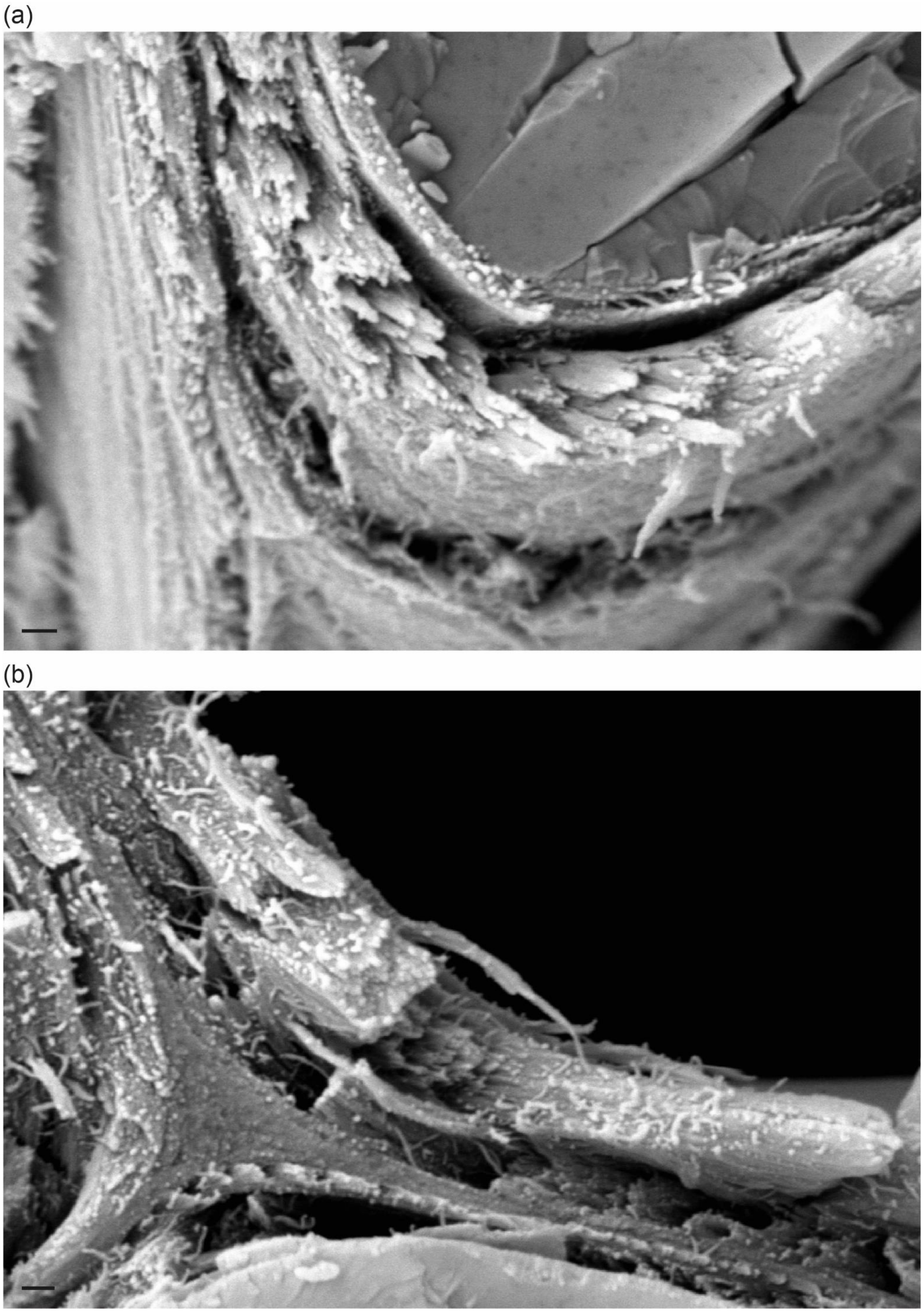

Figure S11. **Further images of cell wall macrofibrils in *4cl1* plants**. Images showing cell walls of *4cl1* (a and b) are provided. Size bars provided correspond to 200 nm on both images.

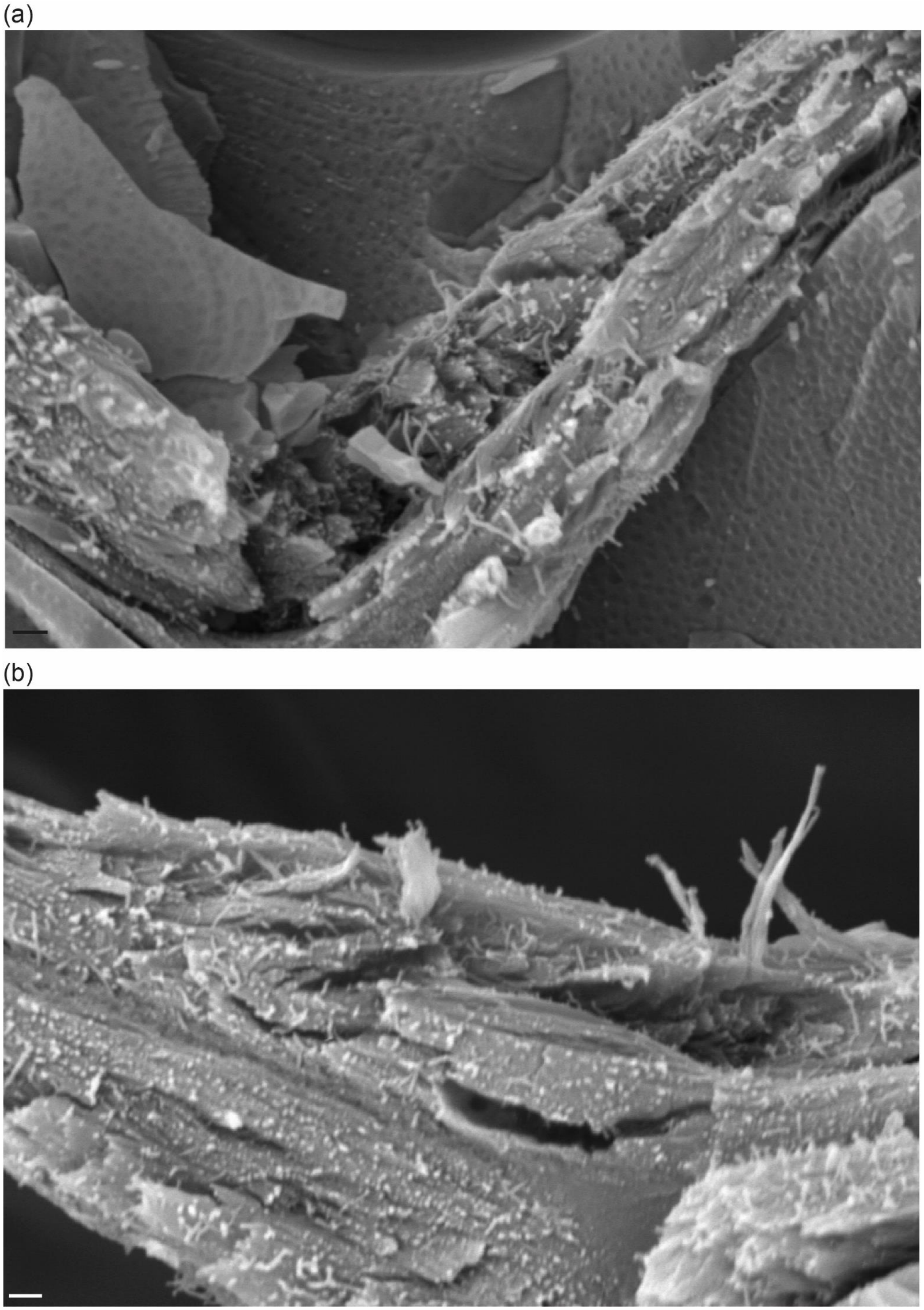

Figure S12. **Further images of cell wall macrofibrils in *lac4* plants**. Images showing cell walls of *lac4* (a and b) are provided. Size bars provided correspond to 200 nm on both images.

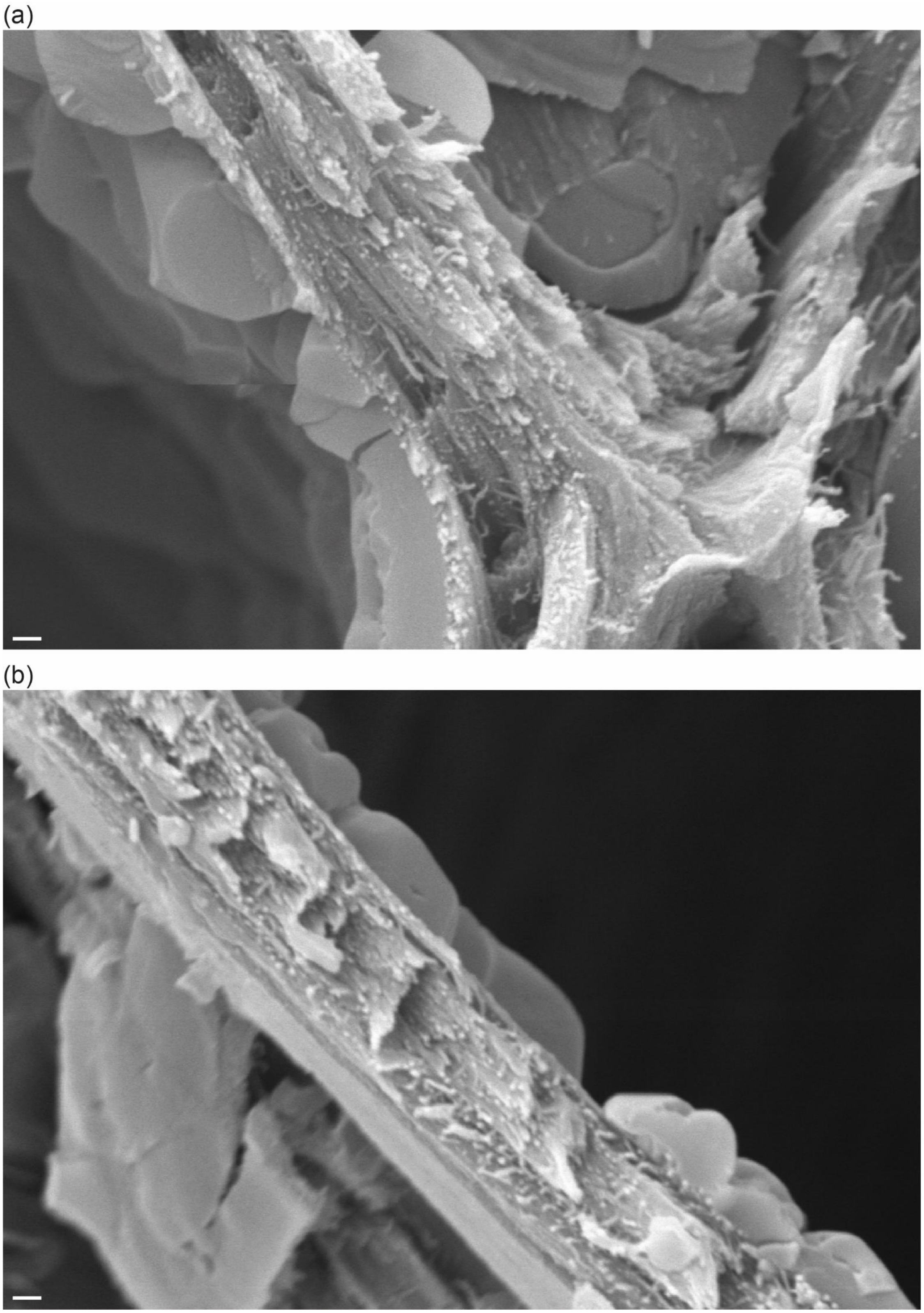

Figure S13. **Further images of cell wall macrofibrils in *csla2/3/9* plants**. Images showing cell walls of *csla2/3/9* (a and b) are provided. Size bars provided correspond to 200 nm on both images.
